# Laetoli, Tanzania: Extant terrestrial mollusc faunas shed new light on climate and palaeoecology at a Pliocene hominin site

**DOI:** 10.1371/journal.pone.0302435

**Published:** 2024-05-16

**Authors:** Peter Tattersfield, Ben Rowson, Christine F. Ngereza, Terry Harrison

**Affiliations:** 1 Department of Natural Sciences, Amgueddfa Cymru–Museum Wales, Cathays Park, Cardiff, United Kingdom; 2 Natural History Museum, National Museums of Tanzania, Arusha, Tanzania; 3 Department of Anthropology, Centre for the Study of Human Origins, New York University, New York, NY, United States of America; Griffith University, AUSTRALIA

## Abstract

Laetoli, Tanzania is one of the most important palaeontological and palaeoanthropological localities in Africa. We report on a survey of the extant terrestrial gastropod faunas of the Laetoli-Endulen area, examine their ecological associations and re-examine the utility of Pliocene fossil molluscs in palaeoenvironmental reconstruction. Standardised collecting at 15 sites yielded 7302 individuals representing 58 mollusc species. Significant dissimilarities were found among the faunas of three broad habitat types: forest, woodland/bushland and open (grassland and scattered, xeric shrubland). Overall, more species were recorded in the woodland/bushland sites than in the forest sites. Open sites were less diverse. Environmental factors contributing most strongly to the separation of habitat types were aridity index and elevation. The results are supplemented with new mollusc data from the Mbulu Plateau south of Lake Eyasi, and compared to the list of species cumulatively recorded from the Ngorongoro area. Some regional variation is apparent and historical factors may explain the absence of some fossil taxa from Laetoli today. Differences in seasonality separated upland forest sites on the Mbulu plateau from those at Lemagurut at Laetoli. Indicator species were identified for each habitat. These included several large-bodied species analogous to the Laetoli Pliocene fossil species that were then used for palaeoenvironmental reconstruction. Based on the estimated aridity index, and adopting the widely used United Nations Environment Programme (UNEP) global climate classification, the four stratigraphic subunits of the Upper Laetolil Beds (3.6–3.85 Ma) would be placed in either the UNEP’s Dry Sub-humid or Semi-arid climate classes, whereas the Upper Ndolanya Beds (2.66 Ma) and Lower Laetolil Beds (3.85-<4.36 Ma) would be assigned to the Humid and Semi-arid climate classes respectively. Pliocene precipitation at Laetoli is estimated as 847–965 mm per year, refining previous estimates. This is close or slightly higher than the present mean annual precipitation, and is likely to have corresponded to a mosaic of forest, woodland and bushland within a grassland matrix consistent with other reconstructions.

## Introduction

**Fig 1:**
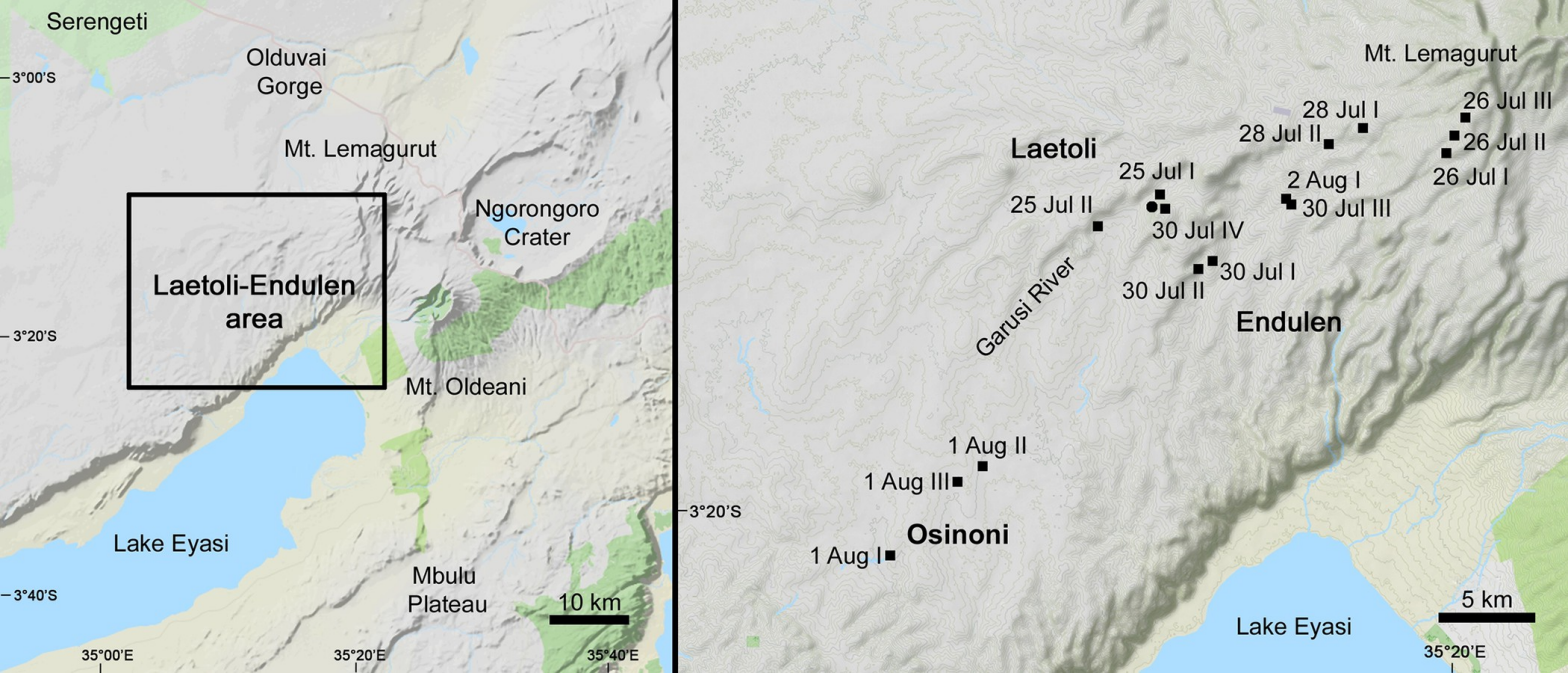
The study area and location of sampling sites. Left: The study area in north-east Tanzania, also showing Ngorongoro and the Mbulu Plateau. Right, sites from which molluscs were collected in 2016. The circle indicates the base camp on the Garusi River. Contains information from OpenStreetMap and OpenStreetMap Foundation, which is made available under the Open Database License.

This paper reports on the first comprehensive and quantitative field study of the terrestrial molluscs (snails and slugs) present in the Laetoli-Endulen (3°S, 35°E) area of Tanzania, which lies on the southern edge of the Serengeti ecosystem and in the Ngorongoro Conservation Area (Fig 1). Its objectives are to describe the mollusc faunas associated with the main vegetation types currently present in the area, from forest to grassland, and to relate variation in the faunas to environmental conditions, including climate. This is relevant to both current conservation and the palaeoenvironmental reconstruction of fossil sites in the area, in which terrestrial molluscs are often abundant [1].

The terrestrial gastropods of Tanzania remain under-studied compared to their freshwater counterparts. The fauna is incompletely described and under-recorded, and the ecology of most species is barely known. Studies on the ecology of the East African land-snail fauna have focussed strongly on the region’s closed forest habitats that support the great majority of the region’s molluscan diversity [2], despite covering less than about 3% of its land area [3]. Evergreen and montane forest faunas have been received the greatest attention (e.g. [4–6]), although the land-snails of other East African forest types have also been studied, including gallery forest [7] and coastal forest and woodland [8–11].

In contrast, and with the notable exception of an account of the molluscan fauna of a Rwandan savanna ecosystem [12], there have been no detailed studies on the faunas associated with East Africa’s other major vegetation types. Knowledge of the faunas of non-forested areas in East Africa has previously been based on anecdotal species lists and literature records; there is no review like that provided for South Africa [13]. Pickford [14], in particular, provides species lists from six simplified East African vegetation categories, ranging from rainforest to desert, and discusses their value for palaeoecological interpretation. Mollusc lists have been published for other East African localities, but these have generally not been collected in a systematic way and are rarely accompanied by detailed information on environmental conditions or vegetation. Pilsbry and Bequaert [15] included species lists from various localities in the Congo basin and neighbouring areas, although their focus was on understanding the biogeographical relationships of the fauna, rather than its ecological associations and determinants.

Laetoli itself is one of the most important palaeontological and palaeoanthropological localities in Africa [16,17 and references therein]. Its Plio-Pleistocene beds are highly fossiliferous for many groups of fauna, providing key biochronological and dating references for other sites on the continent. In particular, Laetoli is celebrated for its remains of the hominins *Australopithecus afarensis* and *Paranthropus aethiopicus*, and for the trails of footprints attributed to individuals of *A*. *afarensis*. The character of the hominin habitats and the palaeoecological setting are thus of global interest, with other fossil groups including terrestrial gastropods (no freshwater Mollusca have been found) having much to offer the environmental reconstruction.

**Fig 2:**
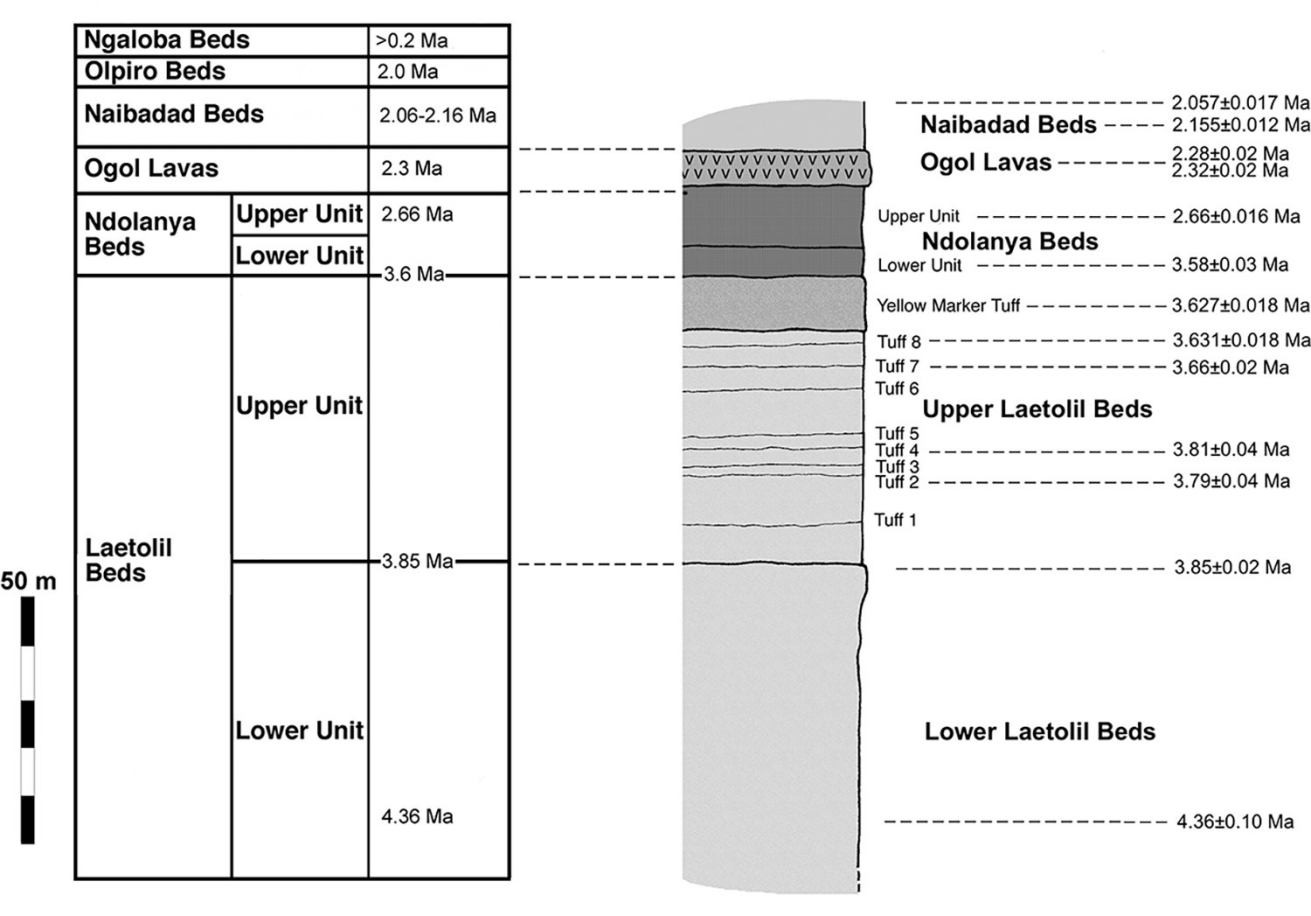
Stratigraphic scheme and geochronology of the main lithological units at Laetoli (adapted from [20]).

No detailed survey of the extant gastropods of the Laetoli area, particularly in non-forest (e.g. woodland and grassland) habitats, has yet been undertaken. Fossil gastropods from Laetoli have previously been studied and interpreted with respect to their most similar extant analogues [1,14,18,19]. However, no systematic attempt has been made to determine whether these faunas can be found living nearby. To date, at least 20 fossil mollusc species have been recorded from the Pliocene beds (Table 1, Fig 2).

**Table 1:** Pliocene fossil molluscs recorded from the Laetolil and Ndolanya Beds [1,14,18,19].

Pliocene fossil Mollusca				Lower Laetolil Beds			Upper Laetolil Beds						Upper Ndolanya Beds	
Family (after [32])	Genus	Species	Authority / source	3.85-<4.36 Ma			3.6–3.85 Ma						2.66 Ma	
				Pickford (1995)[14] (no species names for some taxa)	Verdcourt (1987)[19]	Tattersfield (2011)[1]	Boettger (1947)[18]	Verdcourt (1987)[19]	Tattersfield (2011)[1]				Verdcourt (1987)[19]	Tattersfield (2011)[1]
									Tuffs 0–3	Tuffs 3–5	Tuffs 5–7	Tuff 7—YMT		
Succineidae	*"Succinea"*	sp. A	of Tattersfield (2011)			x		x						
Achatinidae	*Burtoa*	*nilotica*	(L. Pfeiffer, 1861)			x		x	x		x	x		
Achatinidae	*Limicolaria*	*martensiana*	(Smith, 1880)	x	x	x					x			
Achatinidae	*Achatina (Lissachatina)*	*zanzibarica*	Bourguignat, 1879	?	x	x	x		x	x	x		?	
Achatinidae	*Achatina (Lissachatina)*	*fulica hamillei*	(Petit, 1859)				x							
Achatinidae	*Subulona*	*pseudinvoluta*	Verdcourt, 1987				x ^1^	x	x	x	x	x		
Achatinidae	*Pseudoglessula (Kempioconcha)*	cf. *gibbonsi*	(Taylor, 1877)	?				x	x					
Achatinidae	*Kenyaella*	*leakeyi*	(Verdcourt, 1987)				x ^2^	x	x	x	x	x		
Achatinidae	*Kenyaella*	*harrisoni*	Tattersfield, 2011										? ^3^	x
Achatinidae	*Subuliniscus*	sp. A	of Tattersfield (2011)											x
Streptaxidae	*Gulella*	sp. A	of Tattersfield (2011)								x	x		
Streptaxidae	*Streptostele (Raffraya)*	aff. *horei*	aff. *urguessensis* Conn. in Verdcourt (1987)					x			x	x		x
Streptaxidae	*Streptostele*	sp. A	of Tattersfield (2011)								x			
Cerastidae	*Gittenedouardia*	*laetoliensis*	(Verdcourt, 1987)	?		x		x	x	x	x	x		
Cerastidae	*Cerastus*	sp. A	of Tattersfield (2011)			x								
Pupillidae	*Pupoides*	*coenopictus*	(Hutton, 1834)	x										
Hygromiidae	*Halolimnohelix*	*rowsoni*	Tattersfield, 2011									x		
Urocyclidae	*Trochonanina*	sp. A	cf. *obtusangula* Mts. in Verdcourt (1987)					x						x
Urocyclidae	*Trochonanina*	sp. B	cf. *elatior* Mts. in Verdcourt (1987)	?		x		x	x	x	x	x		
Urocyclidae	Urocyclid slug	indet.	spp. A-E in Verdcourt (1987)					x	x	x	x	x		x
**Total species recorded for each bed (slugs treated as 1 species)**				** **		**9**	** **					**16**	** **	**6**

YMT, Yellow Marker Tuff.

^1^ As *Subulona lenta* (Smith), here assigned.

^2^ As *Subuliniscus* sp., here assigned.

^3^ One specimen of uncertain identity, here assigned to *K*. *harrisoni*.

The stratigraphy, lithology, and geochronology of Laetoli are reviewed in Harrison [20]. The Upper Laetolil Beds are particularly rich, with 16 species recorded. The urocyclid slugs were split by Verdcourt [19] into several species, but are considered as a single taxon here since identification of genera and species depends on dissection, and is not possible from the (fossil) shells. Gastropods have been integrated into palaeoecological reviews of the Upper Laetolil Beds [21] and the younger Upper Ndolanya Beds [22]. A small number of terrestrial mollusc taxa is also known from the beds at the nearby Olduvai Gorge, and is considered to reflect a wooded savanna environment during the later Pliocene [23].

Boettger [18] was the first to report fossil land-snail taxa from Laetoli, including from the Upper Laetolil Beds at “Garussi (Vogelfluß)”. His work has sometimes been overlooked but it is possible to assign his taxa to broad strata. He reported that his shells were found ‘without exception enclosed in a coarse-grained, grey-white tuff, which also completely fills the inside of the shells’; this matrix is characteristic of the Pliocene material from the Laetolil Beds studied by Verdcourt [19] and Tattersfield [1], and quite unlike the finer grained sediments associated with Pleistocene material from same general area. Four of Boettger’s [18] gastropods can be referred to taxa subsequently dealt with by Verdcourt [19] and/or Tattersfield [1] (Table 1). The fifth is *Achatina (Lissachatina*) *acuta*, which was subsequently identified by Bequaert [24] as *A*. *(Lissachatina*) *fulica hamillei*. Verdcourt [19] observed that even fresh *Achatina* material can often not be named with confidence, but since both Boettger [18] and Bequaert [24] recognised two separate *Achatina* species in this material it would appear, at least provisionally, that two separate *Achatina (Lissachatina*) species need to be reported from the Laetoli Pliocene. In addition, Boettger [18] recorded *Burtoa* from “Lemagurut Korongo”, an early Pleistocene site nearby.

The floristic communities of a plateau like the Laetoli-Endulen area are complex and structured by soil type, drainage, and anthropogenic influences [25]. This would of course be expected of the snail faunas. Some regional context is also required, because the distribution of different molluscan lineages within East Africa is another powerful structuring factor. Rates of molluscan endemism are high for many individual forest blocks. Turnover and endemism are thought to result partly from historical patterns of movement, isolation and extinction. There are stark discontinuities in the distribution of some of the larger-bodied genera in East Africa, including some known as fossils [26]. The prevailing view that this applies predominantly to forest taxa [2] may be an oversimplification. The best published data on molluscs of the area [27] is in the form of a list for the Ngorongoro volcanic area, with other data confined to the primary taxonomic literature. Other works dealing with records of terrestrial molluscs from this part of Tanzania include von Martens [28], Haas [29], Pfeiffer [30] and the other publications of Verdcourt (listed in [31]). We account for these sources, as well as unpublished mollusc data from forest sites on the Mbulu Plateau (to the south east of Lake Eyasi), to consider the Laetoli fauna in the context of its neighbours.

## Methods

### Survey and identification

Molluscs were surveyed at 15 sites across a range of habitats and elevations in July and August 2016 (Table 2, Fig 1); additional material was also collected from three further locations. All sites were located within an area of roughly 10 x 40km, extending from the elevated Lemagurut area in the north-east to the Osinoni plains in the south-west (Fig 1). The maximum distance between sites was about 37km and the elevational range was about 1000m, ranging from 1580m to 2600m asl.

**Table 2:** Mollusc sampling sites at Laetoli in 2016, and on the Mbulu Plateau in 1998.

Date/code	Site name	Habitat type	Habitat detail	Latitude	Longitude	Elevation (m)	Soil pH
**Main mollusc sampling sites**							
25 Jul 2016 I	Garusi River valley (near camp)	Woodland/bushland	Open riverine woodland on mbuga soils	-3.21	35.22	1800	7.48
25 Jul 2016 II	Garusi River valley (near footprints)	Woodland/bushland	Open riverine woodland and bushland on mixed soils and tuff	-3.22	35.19	1750	-
26 Jul 2016 I	Lemagurut lower forest	Forest	Montane forest	-3.19	35.35	2420	7.08
26 Jul 2016 II	Lemagurut upper forest	Forest	Montane forest	-3.18	35.36	2600	7.58
26 Jul 2016 III	Lemagurut upper forest	Forest	Montane forest	-3.18	35.36	2450	7.13
28 Jul 2016 I	River gorge forest	Woodland/bushland	Woodland/gallery forest	-3.18	35.32	2150	7.59
28 Jul 2016 II	River gorge acacia woodland	Woodland/bushland	Tall, open woodland, acacia	-3.19	35.3	2100	7.12
30 Jul 2016 I	Endulen Yellow-barked acacia	Woodland/bushland	Tall closed riverine woodland, yellow barked acacia	-3.24	35.25	1610	7.45
30 Jul 2016 II	Endulen slope woodland	Woodland/bushland	Open woodland/dense bush	-3.24	35.24	1670	7.49
30 Jul 2016 III	Endulen escarpment woodland	Woodland/bushland	Tall open woodland	-3.21	35.28	1900	7.19
30 Jul 2016 IV	Hill above base camp	Woodland/bushland	Bushland with sparse trees	-3.21	35.22	1850	7.38
1 Aug 2016 I	Osinoni Aloe and Euphorbia xeric scrub	Open	Xeric scrub with scattered short acacia	-3.37	35.09	1620	7.78
1 Aug 2016 II	Osinoni scrubby grassland	Open	Grassland, scatterd sparse scrub	-3.34	35.12	1580	7.87
1 Aug 2016 III	Osinoni ashfall	Open	Xeric grassland, possibly calcrete pan	-3.33	35.13	1600	-
2 Aug 2016 I	Endulen riverine woodland near hospital	Woodland/bushland	Riverine woodland, acacia	-3.21	35.28	1890	7.68
**Mbulu Plateau sites (1998)**							
24 Jun 1998 I	Nou FR, montane forest in valley	Forest	Forest	-4.07	35.47	2100	-
24 Jun 1998 II	Nou FR, montane forest on ridge	Forest	Forest	-4.07	35.47	2300	-
24 Jun 1998 III	Nou FR, montane forest in valley	Forest	Forest	-4.07	35.47	2100	-
26 Jun 1998 I	Hassama Hill FR, forest on plateau top	Forest	Forest	-3.9	35.67	2100	-
26 Jun 1998 II	Hassama Hill FR, woodland and forest	Forest	Forest	-3.9	35.67	2000	-

The 15 sites were distributed across a range of habitat types from forest (evergreen montane, gallery), through various woodland types (riverine, escarpment and mbuga or black cotton soil/vertisol), bushland (sometimes interspersed with open woodland) to open habitats (xeric scrub and grassland) [25]. Vegetation on some of the sites is clearly influenced by anthropogenic factors, especially grazing, wood cutting, and occasional burning by Maasai pastoralists. This has probably caused a reduction in tree cover in some areas, making it more difficult to separate bushland, woodland and forest types. Based on the field-based site classification, three of the sites could be confidently assigned to the evergreen montane forest category (on Lemagurut) and a further three were on open habitats (Osinoni, around the town of Sinoni). The other nine sites were sampled in various types of woodland, forest or bushland, or mosaics of these habitat types. Woodland sites included sites dominated by yellow-barked acacia (*Vachellia xanthophloea*), and sites on black cotton (mbuga) soils.

Local habitat conditions were recorded on each site, the following being estimated by visual assessment:

**Disturbance**: ranked from 1 (no evidence of significant human disturbance) to 5 (intensively disturbed, for example, by stock grazing, cutting of wood or fire). **Tree canopy and understory cover**: percentage cover of tree and understorey vegetation was estimated and then assigned to 20% bands (0–20%, 20–40% etc). **Canopy height:** average tree height, estimated visually across the site. **Tree size:** ‘diameter at breast height’ (dbh) was estimated, and the approximate range and average values recorded to provide a further measure of tree size. **Site inclination:** average slope of the site was estimated visually. **Mean litter depth:** calculated from several estimates taken across the site, including during the mollusc sampling. **Snail microhabitats:** the presence of potential molluscan microhabitats, including dead wood, rock faces, poorly drained areas, was recorded at each site. **Substrate pH:** pH readings, in water, were taken from the sieved litter residue using a Mettler Toledo MP120 pH meter, calibrated using pH 4 and pH 7 buffer solutions. All soils were slightly alkaline—pH 7.08–7.87.

At each site, molluscs were sampled by between three and eight collectors (BR, PT, CN and assistants), yielding a total of 84 samples, each sample being taken from a plot area of c. 1000-2500m^2^. Collection methods were standardised and collection assistants were trained prior to sampling. Edge habitats (e.g. forest boundaries) and localised areas of disturbance were avoided, as far as possible, during sampling. Molluscs were sampled using direct searching (1–2 person hours per collector) and by collecting leaf litter samples (3–8 litres per collector) for subsequent processing. Litter samples were dried and sieved (minimum mesh size 0.5mm), and all molluscs extracted at the Garusi camp and at the National Natural History Museum, Arusha. The samples from all collectors at each site were combined for the purposes of the analysis.

Material was sorted and identified to species or subspecies level at the National Museum of Wales, Cardiff, with reference to the taxonomic literature and other collections from East Africa. Snails were identified using conchological characters and slugs were identified by dissection. Nomenclature and family assignment follow MolluscaBase [32]. Published names could not be assigned to five, minute snail morphospecies (*Truncatellina* SMOOTH; Valloniid sp. e; Valloniid sp. a; *Trachycystis* sp. PT; and *Afroguppya* sp. PT). Apart from *Truncatellina* SMOOTH, these taxa (or very similar ones) have been noted during surveys elsewhere in East Africa (P Tattersfield, B Rowson and CF Ngereza unpublished). However, their taxonomic status is currently unclear and it is likely that some or all may be undescribed. The specimens are in the collections of the National Museums of Tanzania, with representative samples at the National Museum of Wales. The research presented here was authorized by the Tanzania Commission for Science and Technology (COSTECH) (Research Permit 2016-150-NA-95-175) and the Antiquities Division of Tanzania (Excavation/Collectors Licence No. 04/2016/2017). It does not require IRB or IACUC approval.

For added context, the fauna was compared with that collected using similar methods in two forest reserves on the Mbulu Plateau in 1998 (P Tattersfield, MB Seddon and CF Ngereza, unpublished). The Mbulu sites (Table 2) lie to the south of Lake Eyasi and are roughly 100km to the south-south-east of Laetoli. The specimens collected on the Mbulu Plateau were sorted and identified with reference to the Laetoli material. The lists of taxa recorded from Laetoli and Mbulu were also compared with those from the Ngorongoro area as reported by Verdcourt [27], and the extent of overlap between the three areas was estimated.

**Diversity and abundance.** Completeness of inventory for each site was assessed with reference to the guidelines provided by [33] for land-snail sampling. EstimateS 9.1.0 [34] was used to calculate the Chao-1 species richness estimator. The mean number of specimens per collector was used as an approximate expression of snail abundance for each site.

#### Habitat comparisons and indicator species

Mollusc community richness and abundance data were analysed with a variety of methods to explore associations amongst the sites, and to identify species or/and species groups that might perform as habitat indicators. Except where noted, analyses were performed using PAST software [35]. Singletons and species present at only one site were omitted from most analyses because they are unlikely to contribute to the interpretation of species composition, or to represent reliable habitat indicators. The removal of these species resulted in the inclusion of 48 species in the analyses.

Species composition of the Laetoli faunas was analysed using Detrended Correspondence Analysis (DCA), with log transformed abundance data. Correspondence Analysis (CA), based on binary data, was used to incorporate the Mbulu and fossil data. These techniques enabled us to explore any clustering of sites and the main gradients in the data.

Analysis of similarities (ANOSIM) based on the Bray-Curtis index was used to test the statistical significance of any difference between the mollusc communities of broad habitat types.

We used Two Way Indicator Species Analysis (TWINSPAN, using TWINSPAN for Windows version 2.3 [36] to identify groups of sites containing similar faunas, and groups of species that may be useful as habitat indicators. Presence-absence data, with singletons and species present at only one site omitted, were used since its main purpose was to identify groups of species that are indicative by virtue of their presence or absence of particular habitat conditions. TWINSPAN is a method for classifying species and samples, producing an ordered two-way table of their occurrence. The process of classification is hierarchical; samples are successively divided into categories, and species are then divided into categories on the basis of the sample classification. Minimum group size for division and maximum number of division levels were set at 5 and 3 respectively.

#### Relationships with environmental variables

Relationships between the mollusc fauna and current environmental variables, also incorporating the Mbulu Plateau forest site data, were explored using correlation and canonical correspondence analysis (CCA). Snail abundance data were log transformed, and singletons and species only recorded on one site were omitted.

Correlation analysis was used to explore relationships between snail richness (Chao-1) and abundance, and the local environmental and habitat variables. In order to standardise for variations in the collecting intensity, snail abundance was expressed as the number of individuals per collector.

Canonical correspondence analysis (CCA) was used to explore the relationship between wider climate conditions and snail faunas. CCA is a multivariate method that assists analysis of the relationships between biological assemblages of species and their environment. It is a direct gradient method and extracts synthetic environmental gradients from ecological data sets, the objective being to succinctly describe and visualise the differential habitat preferences of taxa via an ordination diagram.

Environmental data used in the CCA included altitude, plus the following variables that were obtained, for each site, from third party sources online. An Aridity Index (AI_ET0) [37], which represents the ratio between precipitation and reference (potential) evapotranspiration ET0, thus precipitation availability over atmospheric water demand (aggregated on annual basis). Under this formulation, Aridity Index values increase for more humid conditions and decrease with more arid conditions. Following Trabucco and Zomer [37], AI_ET0 has been multiplied by 10,000 in order to express the measure in integers. The CCA also included eleven bioclimatic variables from the Worldclim database [38], namely: BIO1 = Annual Mean Temperature; BIO2 = Mean Diurnal Range (Mean of monthly [maximum–minimum temperature]); BIO3 = Isothermality (BIO2/BIO7) (x 100); BIO4 = Temperature Seasonality (standard deviation x 100); BIO7 = Temperature Annual Range (BIO5-BIO6); BIO12 = Annual Precipitation; BIO15 = Precipitation Seasonality (Coefficient of Variation); BIO16 = Precipitation of Wettest Quarter; BIO17 = Precipitation of Driest Quarter; BIO18 = Precipitation of Warmest Quarter; BIO19 = Precipitation of Coldest Quarter.

In turn the analysis was extended, using Correspondence analysis (CA), to include the extant species and the fossil molluscs of the Laetoli Beds, treating each bed as a site without environmental data and tentatively matching extant to fossil mollusc species. The analysis was based on presence-absence data and was restricted to species that occurred in at least one fossil bed and/or at least one sampling site. This allowed us to interpret the ecological relationships between extant and fossil faunas, and thus the dominant bioclimatic factor(s) for the fossil communities.

The site-based environmental factors (habitat structure, disturbance etc) were used to interpret the habitat groupings identified using TWINSPAN, and in correlation analysis to explore relationships with snail abundance and species richness.

## Results

### Diversity and abundance

A total of 7302 individual molluscs representing 58 species (or subspecies) was recovered from the 15 sites (S1 Table).

The number of species per site ranged from 6–24 (mean 15.2, SD 6.0), and the total number of individuals from 39–1544 (mean 487, SD 512). The mean number of individuals per species ranged from 4.0–64.3 (mean 26.6, SD 21.0) (Table 3). Samples from five of the 15 sites did not meet minimum recommendations for land-snail sampling, where the objective is to compile inventories [33], so faunal lists for these sites in particular may not be complete. These were the sites with the lowest number of individuals–in acacia woodland (30.IV, 2.I), riverine woodland (25.I, 25.II) and the Osinoni ashfall site (1.III). Overall, the number of species recorded increases with the number of individuals found (r^2^: 0.676, p<0.001), indicating that caution is required when interpreting the variation in species richness because it may be influenced by sample size. We therefore calculated the Chao-1 (bias corrected) estimator of species richness (Table 3) using EstimateS [34], and used this for species richness comparisons between sites and with environmental variables. The Chao-1 estimator is based on the number of species that are represented in a sample by one (singletons) or two (doubletons) individuals. Although it was derived as a lower bound of species richness [39,40] have subsequently verified for many datasets that it is relatively good point estimator of the total species richness of a community, thereby justifying its use as a species richness estimator. The Chao-1 estimator indicated that all recorded inventories were at least 71% complete (Table 3), with all save three sites exceeding 87% completeness. The three sites where inventories may be less complete were the riverine woodlands (sites 25.II (71% complete) and 2.1 (77%)) and the Lemagurut upper forest (site 26.II (76%)). Based on Chao-1, between 3 and 6 species may have gone undetected at each of these sites.

**Table 3:** Number of species and individuals found at the main Laetoli sites in 2016, with Chao-1 richness estimates.

Date/code	Site name	Habitat type	No. collectors	No. species recorded	Chao-1 richness	% completeness	Total no. individuals	Mean individuals per collector	Mean individuals per species
25 Jul 2016 I	Garusi River valley (near camp)	Woodland/bushland	7	12	12.3	98	55	8	4.6
25 Jul 2016 II	Garusi River valley (near footprints)	Woodland/bushland	7	11	16.9	71	58	9	5.3
26 Jul 2016 I	Lemagurut lower forest	Forest	7	19	22.3	99	981	141	51.6
26 Jul 2016 II	Lemagurut upper forest	Forest	4	19	25	76	996	240	52.4
26 Jul 2016 III	Lemagurut upper forest	Forest	3	18	18	100	421	140	23.4
28 Jul 2016 I	River gorge forest	Woodland/bushland	8	21	26	88	1510	184	71.9
28 Jul 2016 II	River gorge acacia woodland	Woodland/bushland	7	21	24	100	1534	220	73
30 Jul 2016 I	Endulen Yellow-barked acacia	Woodland/bushland	7	19	21	91	274	39	14.4
30 Jul 2016 II	Endulen slope woodland	Woodland/bushland	6	20	20	100	448	78	22.4
30 Jul 2016 III	Endulen escarpment woodland	Woodland/bushland	6	8	9	89	234	47	29.3
30 Jul 2016 IV	Hill above base camp	Woodland/bushland	3	7	8	88	39	13	5.6
1 Aug 2016 I	Osinoni Aloe and Euphorbia xeric scrub	Open	7	15	15.5	97	220	31	14.7
1 Aug 2016 II	Osinoni scrubby grassland	Open	6	12	15	87	377	63	31.4
1 Aug 2016 III	Osinoni ashfall	Open	3	6	6	100	96	32	16
2 Aug 2016 I	Endulen riverine woodland near hospital	Woodland/bushland	6	10	12.9	77	39	7	3.9

The three montane forest sites returned totals of 18 to 22 species, with Chao-1 estimates of 18–25 species. There was considerable variation in species richness within the woodland/bushland group, possibly reflecting the rather wide variation in habitat structure in this group. However, four of these sites, 28.I, 28.II, 30.I and 30.II, returned notably higher richness (19–24 species or 20–26 Chao-1) than the other five woodland/bushland sites (7–12 species or 8–16.9 (Chao-1)). Sites sampled on the 28th and 30th July were geographically close to each other, perhaps suggesting a degree of spatial autocorrelation.

Of the 58 species recorded during the survey, 49 species (85%) were found in at least one of the woodland/bushland sites (Table 4), perhaps also reflecting the breadth of habitat conditions in this habitat type. Only 26 species (45%) were recorded in at least one of the forest sites, and 18 (31%) in at least one of the open sites. As expected, open habitat sites also had the lowest abundance (Table 4) and the lowest species richness (6–15 species/6-15.5 (Chao-1)). Mollusc abundance (as measured by mean specimens returned per collector, Table 3) was highest in the montane forest sites on Lemagurut (26.I, 26.II and 26.III), and also in the river gorge gallery forest/woodland sites (28.I and 28.II); 71% of the specimens collected from the woodland/bushland sites derived from these two sites.

**Table 4:** Total abundance for each habitat type, with overall number of species and Chao-1 richness estimates.

Habitat type	No. sites	Total abundance (no. individuals recorded)	Overall no. species recorded	Overall Chao-1 richness
Forest	3	2365	26	27.5
Woodland/bushland	9	4243	49	49
Open	3	694	18	20.2
All	15	7302	58	58.7

### Habitat comparisons and indicator species

There was substantial variation in the species found across sites and habitat types. 26 species (44.8%) were found only in a single habitat type: 5 species (8.6%) only in forest, 17 (29.3%) only in woodland/bushland, and 4 (6.9%) only in open habitats. Only three species (5.2% of the total recorded) were found in all three habitat types. Of the remaining 29 species (50%), 18 (31%) were recorded in both forest and woodland/bushland, and 11 (19%) were found in both woodland/bushland and open habitats. Apart from the 3 species present in all habitat types, no species were common to both forest and open habitats.

**Fig 3:**
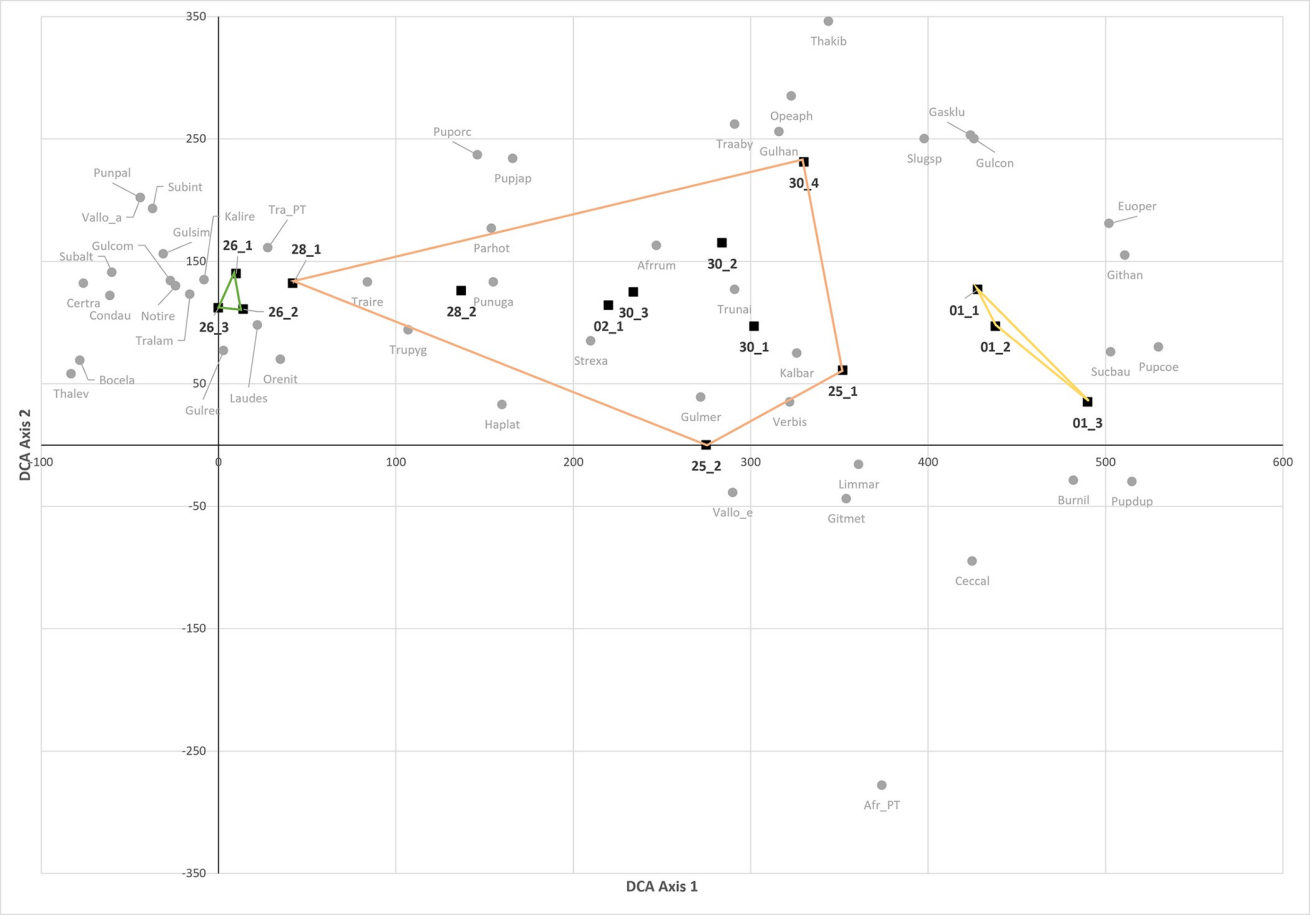
Axes 1 and 2 of the Detrended correspondence analysis (DCA) of sampling sites and mollusc species. Singletons and species occurring on only one site were excluded. Polygons enclose all sites of the same habitat type, as classified in the field, green (forest (3 sites); purple–woodland/bushland (9 sites); yellow–open (3 sites). Eigenvalues–Axis 1: 0.748, Axis 2: 0.169.

Based on abundance data, the mollusc communities were clearly separated by detrended correspondence analysis (DCA, Fig 3) into clusters corresponding to the broad habitat types. The forest and open sites were each tightly clustered, with those for woodland/bushland more loosely so. On axis 1 of the DCA, sites 28.I and 28.II are positioned in the main cluster of woodland/bushland sites but near to the forest group, suggesting that these faunas have a closer affinity with the forest sites than the woodland/bushland group to which they were assigned in the field.

Analysis of similarities (ANOSIM) based on the Bray-Curtis index (composition and abundance of species) identified significant dissimilarity amongst the three broad habitat types. The overall R value (where a value of 1 indicates complete dissimilarity) amongst the three groups was 0.580 and highly significant (P<0.001). Pairwise comparisons indicated high and significant dissimilarity between open and both woodland/bushland and forest categories (Table 5). The R of one value between the forest and open sites was not significant, presumably because of the low sample size (total six sites).

**Table 5:** ANOSIM R (top right) and P (lower left) values, based on Bray-Curtis coefficient, for pairwise comparisons between the three habitat categories.

	Forest	Woodland/bushland	Open
**Forest**	-	0.354	1
**Woodland/bushland**	0.04*	-	0.649
**Open**	0.1	0.01*	-

Data: abundance omitting singletons and species represented at only one site. P-values are uncorrected; *, P < 0.05.]

**Fig 4:**
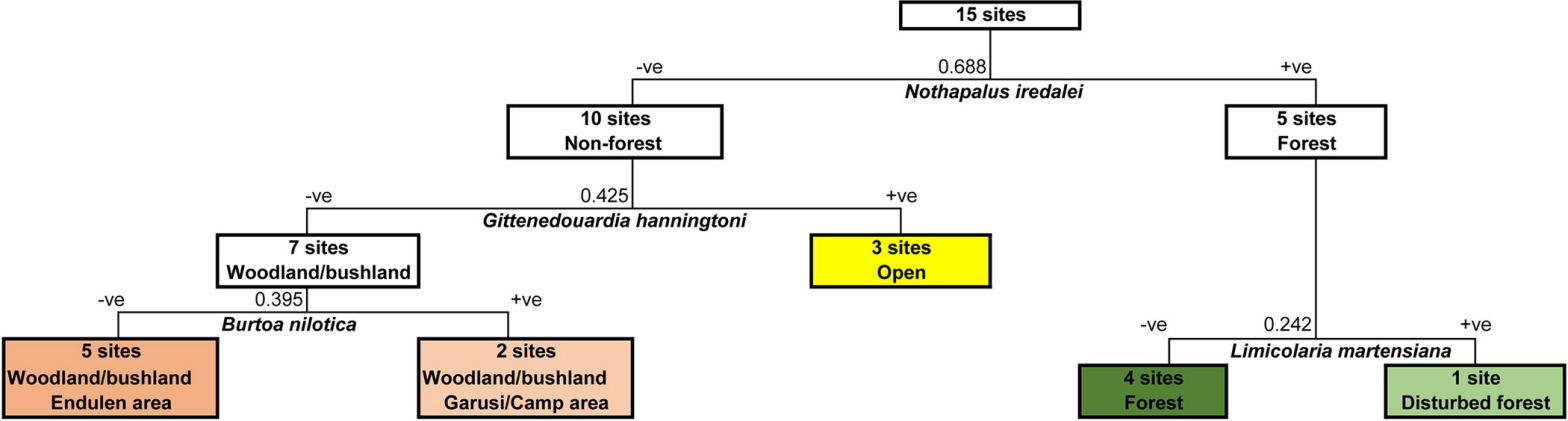
Division of the 15 sites into five groups (coloured) created by TWINSPAN.

The first three levels of the TWINSPAN analysis identified five habitat groupings that were classified by four indicator species. TWINSPAN first undertakes an ordination of the samples and then uses this to obtain a classification of the species according to their associations; each group is defined by the presence or absence of one or more indicator species. Fig 4 shows a tree with the division of the sites and the indicator species, and Table 6 shows this classification in an ordered two-way table that expresses the species’ synecological relations as succinctly as possible. The five groupings corresponded to our open habitat type, plus the forest and woodland/bushland habitat types, both of which were divided into two groups.

**Table 6:** Ordered two-way classification of TWINSPAN results also identifying site groupings.

Species	26 Jul I	26 Jul II	26 Jul III	28 Jul I	28 Jul II	30 Jul I	30 Jul II	30 Jul III	2 Aug I	24 Jul I + 30 Jul IV	25 Jul I	25 Jul II	1 Aug I	1 Aug II	1 Aug III
*Libobocageia elata*	1	1	1												
***Nothapalus iredalei/suturalis***	**1**	**1**	**1**	**1**	**1**	** **	** **	** **	** **	** **	** **	** **	** **	** **	** **
*Subuliniscus alticolus*	1	1	1	1											
*Subulina intermedia*	1			1											
*Gulella commoda*	1	1	1	1	1										
*Cerastus trapezoidea masaica*	1	1	1												
*Conulinus daubenbergi*	1	1	1	1											
*Vallonid spiral apex sp*. *a*	1			1											
*Punctum pallidum*	1			1											
*Trachycystis lamellifera*	1	1	1	1	1										
*Kaliella iredalei*	1	1	1	1	1										
*Trochonanina cf*. *levistriata*		1	1												
*Oreohomorus nitidus*	1	1	1	1	1							1			
*Gulella cf*. *rectangularis*	1	1	1	1	1							1			
*Gulella simplicima*	1		1	1	1										
*Lauria cf*. *desiderata*	1	1	1	1	1										
*Trachycystis sp*. *PT*	1	1	1	1	1										
*Truncatellina pygmaeorum*		1			1										
*Punctum ugandanum*				1	1	1	1								
*Trachycystis iredalei*	1		1	1	1				1						
*Pupisoma (Psychopatula) dioscoricola*	1			1	1		1		1						
*Salpingoma harpula*				1	1		1								
*Paralaoma servilis*	1	1	1	1	1	1	1	1	1					1	1
*Streptostele exasperata*	1	1	1	1	1	1	1		1	1	1	1	1	1	
*Afroguppya rumrutiensis*					1	1	1							1	
*Gulella meruensis*				1	1	1	1	1	1		1	1	1		
*Truncatellina naivashaensis*					1	1	1	1			1		1	1	
*Haplohelix lateaperta*	1	1	1	1	1	1		1	1			1			
*Trachycystis abyssinica*	1				1	1	1	1		1	1		1	1	
*Opeas aphantum*							1	1	1	1	1				
*Silvigulella hanangi*						1	1								
*Gittenedouardia metula*						1	1			1	1	1			
*Vertigo bisulcata*						1	1		1		1	1			
*Vallonid spines/lamellae sp*. *e*						1			1						
*Afroguppya sp*. *PT*											1	1			
*Kaliella barrakporensis*						1	1								
*Slug plate (gen*. *and sp*. *indet*.*)*						1		1		1	1		1		
*Thapsia cf*. *kibonotoensis*							1			1					
***Limicolaria martensiana catharia***	** **	** **	** **	** **	**1**	**1**	**1**	** **	** **	** **	** **	**1**	**1**	**1**	** **
*Gastrocopta klunzingeri*						1	1			1	1		1	1	
*Cecilioides callipeplum*						1						1	1		
*Gulella consociata*							1		1				1		
*“Succinea” baumanni*						1							1	1	1
***Burtoa nilotica giraudi***	** **	** **	** **	** **	** **	** **	** **	** **	** **	** **	**1**	**1**	**1**	**1**	**1**
*Euonyma percivali*													1	1	
***Gittenedouardia hanningtoni***	** **	** **	** **	** **	** **	** **	** **	** **	** **	** **	** **	** **	**1**	**1**	**1**
*Pupilla duplicata*											1	1		1	1
*Pupoides coenopictus*													1	1	1
	** **	** **	** **	** **	** **	**Endulen sites**					**Garusi sites**		** **		
	**Forest**				**Disturbed forest**		** **	** **	**Woodland/bushland (group 2a)**		** **	** **	**Open (group 2b)**		
	**Forest (group 1)**					**Non-forest (group 2)**									

Indicator species identified are given beneath each branch and ordination eigenvalues to left of the nodes.

Indicator species shown in bold.

Table 7 shows mean values of various site characteristics for each of the five groups. These include site specific variables including soil pH and vegetation structure (canopy cover, tree size etc), and wider climatic variables, as estimated by the aridity index AI_ET0 and BIOCLIM variables.

**Table 7:** Mean values of species richness, abundance and environmental and habitat variables in the five TWINSPAN groups. Note that some environmental data were unavailable for some sites.

	Forest	Disturbed forest	Woodland/bushland (Endulen)	Woodland/bushland (Garusi)	Open
No sites	4	1	5	2	3
Total Species	30	24	27	16	17
Species/site	19.3	21.0	12.8	11.5	11.0
Individuals/site	977.0	1534.0	206.8	56.5	231.0
Soil pH	7.3	7.1	7.4	7.5	7.8
Elevation (m asl)	2470.0	2098.0	1747.4	1755.0	1590.3
Aridity Index AI_ET0	8148.0	6387.0	4878.8	4775.0	4016.0
Slope (degrees)	16.7	20.0	14.5	16.3	1.3
Litter depth (cm)	3.3	2.0	1.8	1.3	0.7
Canopy cover (%)	75.0	40.0	33.0	20.0	2.5
Understorey cover (%)	45.0	80.0	37.0	50.0	0.8
Tree height (m)	21.7	16.0	13.0	7.5	4.0
Tree dbh typical (cm)	90.0	60.0	33.8	30.0	5.0
Tree dbh max (cm)	195.0	110.0	57.5	50.0	-
Disturbance (1 low to 5 high)	1.7	3.0	3.5	3.0	1.7
BIO1 = Annual Mean Temperature	130.3	158.0	181.4	183.5	192.0
BIO2 = Mean Diurnal Range	88.3	94.0	100.0	100.5	103.0
BIO3 = Isothermality (BIO2/BIO7) (×100)	66.0	68.0	68.6	68.5	70.0
BIO4 = Temperature Seasonality	1250.5	1261.0	1333.2	1295.5	1300.0
BIO7 = Temperature Annual Range	132.5	138.0	145.0	145.5	146.0
BIO12 = Annual Precipitation	1039.5	946.0	858.6	854.0	838.7
BIO15 = Precipitation Seasonality	72.3	78.0	83.0	84.0	83.3
BIO16 = Precipitation of Wettest Quarter	463.8	452.0	439.6	440.5	427.0
BIO17 = Precipitation of Driest Quarter	34.8	19.0	10.2	9.0	10.0
BIO18 = Precipitation of Warmest Quarter	372.3	452.0	391.2	389.5	427.0
BIO19 = Precipitation of Coldest Quarter	43.3	23.0	14.0	12.0	14.0

The five groups can be interpreted in relation to their mollusc faunas, and to their associations with habitat and climatic variables. Table 8 identifies the species that are confined to or strongly associated with the TWINSPAN groups, based on the mean number of specimens recorded per site. These comprise a set of 20 species that are associated with the forest sites and a further 18 species that are confined to or associated with non-forest habitats. Included in this latter category are eight species associated with woodland/bushland habitats and five that have an affinity with the open sites; the remaining five species occur in both woodland/bushland and open sites. Ten species do not show a clear association with any of the main TWINSPAN groups.

**Table 8:** List of all species showing strong associations with each of the five TWINSPAN groups, and those with no clear association. Data are mean number of specimens per site.

			TWINSPAN site groupings				
			Forest (group 1)		Non-forest (group 2)		
**Species confined to or strongly associated with:**			**Forest (other)**	**Forest (disturbed)**	**Woodland/bushland (group 2a)**		**Open (group 2b)**
			** **	** **	**Endulen sites**	**Garusi sites**	** **
			**4 sites**	**1 site**	**5 sites**	**2 sites**	**3 sites**
**Forest (group 1)**			** **	** **	** **	** **	** **
		*Trochonanina* cf. *levistriata*	3.3				
		*Kaliella iredalei*	102.3	24.0			
		*Trachycystis* sp. PT	17.0	17.0			
		*Trachycystis lamellifera*	81.8	15.0			
		*Punctum pallidum*	4.5				
		Vallonid spiral apex sp. a	5.5				
		*Lauria* cf. *desiderata*	3.3	7.0			
		*Truncatellina pygmaeorum*	0.3	2.0			
		*Conulinus daubenbergeri*	16.5				
		*Cerastus trapezoidea masaicus*	47.8				
		*Gulella commoda*	88.5	7.0			
		*Gulella simplicima*	3.3	1.0			
		*Subulina intermedia*	4.8				
		*Subuliniscus alticolus*	23.0				
		*Nothapalus iredalei / suturalis*	106.0	10.0			
		*Liobocageia elata*	72.0				
		*Trachycystis iredalei*	4.5	7.0	0.4		
		*Gulella cf*. *rectangularis*	42.8	7.0		0.5	
		*Oreohomorus nitidus*	128.5	116.0		0.5	
		*Punctum ugandanum*	66.3	59.0	2.8		
**Non-forest (group 2)**			** **	** **	** **	** **	** **
		*Cecilioides callipeplum*			0.2	0.5	0.7
		*Burtoa nilotica giraudi*				5.0	5.3
		*Slug plate* (gen. & sp. indet.)			0.6	0.5	0.3
		*Gastrocopta klunzingeri*			15.4	1.0	11.7
		*Gulella consociata*			1.8		7.3
	**Woodland/bushland (group 2a)**		** **	** **	** **	** **	** **
		*Thapsia cf*. *kibonotoensis*			6.2		
		*Kaliella barrakporensis*			4.6		
		*Afroguppya* sp. PT				2.0	
		Vallonid spines/lamellae sp. e			0.6		
		*Vertigo bisulcata*			36.2	1.0	
		*Gittenedouardia metula*			4	10.0	
		*Silvigulella hanangi*			2		
		*Opeas aphantum*			4.8	1.5	
	**Open (group 2b)**		** **	** **	** **	** **	** **
		*Pupoides coenopictus*					19.3
		*Pupilla duplicata*				2.0	26.7
		*Gittenedouardia hanningtoni*					34.0
		*Euonyma percivali*					1.0
		*“Succinea” baumanni*			0.4		112.0
**No clear association (no group)**			** **	** **	** **	** **	** **
		*Pupisoma harpula*	0.3	7.0	0.4		
		*Haplohelix lateaperta*	6.3	24.0	24.2	5.0	
		*Pupisoma dioscoricola*	4.3	4.0	2.0		
		*Paralaoma servilis*	102.8	388.0	27.4		1.0
		*Streptostele exasperata*	23.0	17.0	15.6	16.0	1.7
		*Afroguppya rumrutiensis*		281.0	6.4		0.3
		*Trachycystis abyssinica*	0.5	6.0	5.8	1.0	1.0
		*Limicolaria martensiana catharia*		8.0	2.8	1.5	3.3
		*Gulella meruensis*	0.3	8.0	8.8	6.0	0.3
		*Truncatellina naivashaensis*		505.0	31.4	3.0	4.7

The first division (Fig 4) is based on the presence/absence of *Nothapalus iredalei/suturalis*. This separates the 15 sites into a smaller group of five sites that contain *N*. *iredalei/suturalis* and 10 others that do not. It can be interpreted as separating sites supporting forest faunas from the others that contain faunas associated with woodland, bushland or open habitats. In addition to the presence of *N*. *iredalei/suturalis*, these forest faunas support much richer faunas and higher levels of snail abundance than the non-forest faunas (Table 7). Although the Laetoli fossil faunas contain a number of achatinid genera of comparable shell size (Table 1), none of these appears to belong to *Nothapalus* as currently circumscribed.

### Classification of forest habitats

The forest group contains the three closed canopy afromontane forest sites on Lemagurut (26.I, 26.II and 26.III), plus two sites along a drainage ravine (28.I and 28.II) about 5km to the west of the Lemagurut sites. The two ravine sites were classified in the field as woodland/ gallery forest, but TWINSPAN, based on their mollusc faunas, supports the DCA analysis (Fig 3) and places these sites closer to the forest group than the woodland/bushland sites. As would be expected, sites in the forest group have much higher canopy and understorey percentage cover, deeper litter and taller and larger trees than the non-forest group (Table 7). The forest sites are typically at higher elevation, on steeper gradients and experience higher annual precipitation and lower aridity levels than the non-forested sites, although mean soil pH and disturbance levels do not appear to differ appreciably (Table 7).

In terms of faunal composition (Table 8), the forest group is characterised by very small or minute species that comprise 50% of the recorded fauna. Many of these live in the leaf litter on the forest floor (for example, *Trachycystis* (Charopidae) and *Punctum* (Punctidae) spp.) although others such as *Lauria* cf. *desiderata* are probably associated with the leaves of ferns and herbs. Cerastids (*Conulinus*, *Cerastus*) and moderate sized achatinids (*Oreohomorus*, *Subuliniscus*, *Liobocageia*) are the largest species associated with the forest habitats. A fossil species of *Cerastus* is known from the Lower Laetolil Beds and a fossil *Subuliniscus* has been reported from the Upper Ndolanya Beds (Table 1). Very large shelled achatinids (*Achatina*, *Limicolaria*, *Burtoa*) are absent or very sparse in the forest sites (although see discussion regarding *Limicolaria* below).

At the second division level, TWINSPAN separates ravine site 28.II from the other four forest sites based on the presence of the indicator species *Limicolaria martensiana* on 28.II. Interpretation is necessarily tentative because of the small number of sites, but the climatic variables indicate that this site experiences a less humid climate (AI_ET0) with lower annual precipitation (BIO12) than both ravine site 28.I and the three Lemagurut forest sites. Site 28.II’s fauna may thus be regarded as intermediate between the forest and the woodland/bushland habitats included in the study. Elsewhere in this study, *L*. *martensiana* was found in 40% of sites in the non-forest group. It is a synanthropic species [14], often being associated with disturbed areas in East Africa and occurring frequently in cultivated areas (shambas). Its presence on site 28.II may thus, at least in part, reflect anthropogenic factors. This is supported by the qualitative index of disturbance, which was assessed to be higher on site 28.II than in the other forest sites. Compared with the other four forest sites, mean tree canopy cover is lower and understorey cover higher on Site 28.II, possibly as a result of the removal of larger trees and consequent increase in a dense shrubby understory. The fauna of Site 28.II also appears impoverished relative to the other forest sites, lacking several characteristic forest species that were abundant on other forest sites such as *Cerastus trapezoidea*, *Conulinus daubenbergi*, *Subuliniscus alticolus* and *Liobocageia elata*.

Three of the species that do not show any clear association with any of the TWINSPAN groups (Table 8) occur at very high abundance in the ravine forest site 28.II. Two of these, both minute litter-dwelling species (*Afroguppya rumrutiensis* and *Truncatellina naivashaensis*), were recorded in other woodland/bushland and open sites but not in forest sites, so their high abundance on site 28.II reinforces its affinity with the woodland/bushland fauna. The other abundant species on 28.II, *Paralaoma servilis* (also a minute litter dweller), was recorded on 11 of the 15 sites so it appears to have a rather broad habitat tolerance, although it was present in much higher numbers on the forest sites. In the present study, this species was initially recorded under the name *P*. *hottentotum* (Melvill and Ponsonby, 1891), a species synonymised with *P*. *servilis* [41].

#### Classification of non-forest habitats

TWINSPAN divides the 10 non-forest sites into a group of three sites with faunas associated with open, more xeric habitats (the xeric scrub at site 01.I and open grassland at sites 01.II and 01.III), and a group of seven sites with woodland/bushland habitats. The division is based on the presence of *Gittenedouardia hanningtoni*, which is restricted to the group of three open sites. A fossil species of *Gittenedouardia* is present in many of the Upper and Lower Laetolil Beds (Table 1).

Sites in the open group have slightly higher soil pH (associated with the presence of unconsolidated soda rich ash) and are situated on level ground at lower elevations. They are more arid, and receive less precipitation and have higher mean temperatures than sites in any of the other TWINSPAN groups (Table 7). In terms of habitat structure, they are characterised by a scarcity of woody vegetation. Three species are confined to these sites—*Gittenedouardia hanningtoni*, *Pupoides coenopictus* and *Euonyma percivali*, although the last is represented by only 3 specimens. A further two species, *Pupilla duplicata* and ‘*Succinea’ baumanni* are strongly associated with these habitats (Table 8).

The larger group of seven woodland/bushland sites do not support *G*. *hanningtoni* and are characterised by rather low shrubby vegetation, with intermediate levels of canopy cover and low tree height and size. They also suffer from higher levels of disturbance, especially grazing and wood cutting. In terms of associated species, *Gittenedouardia metula*, *Opeas aphantum* and *Vertigo bisulcata* were restricted to and frequently recorded in this group, all being present on five of the seven sites.

TWINSPAN identifies a further division of the seven woodland/bushland sites based on the occurrence of *Burtoa nilotica*. The two sites supporting this huge achatinid are in the Garusi area (the ‘Garusi’ group) whereas four of the other five sites are in the Endulen area (the ‘Endulen’ group), the remaining site being close to the base camp near Garusi. Both Garusi sites (25.I and 25.II) support *Burtoa*, but otherwise it was only recorded at the three open sites at Osinoni (01.I, 01.II and 01.III), thus perhaps indicating that the snail fauna is experiencing less humid and more xeric conditions within the Garusi group. However, the climate variables (Table 7) do not reveal any major differences in climate. On average, the Endulen group sites are associated with slightly greater levels of precipitation and less arid conditions, but these differences are very small, at least compared with the range across the whole study area. It seems unlikely that climate alone can account for differences in the faunas. In contrast, there are more marked differences in habitat structure (Table 7), possibly caused by differences in anthropogenic use (e.g. grazing, removal of wood, burning), or by large mammalian herbivores. Overall, the Endulen sites are typically situated on less sloping land, with larger trees and taller and denser canopies and understories, and deeper litter layers, than the Garusi sites. The fauna of the Garusi sites appears to be rather impoverished, with a total of 16 species being recorded compared with 27 in the Endulen group. This may be related to differences in vegetation structure, the taller and denser conditions on the Endulen sites providing more humid, stable and abundant mollusc microclimates, with deeper litter layers, thus possibly providing niches for a wider range of species. However, sample sizes were much smaller on the two Garusi sites so there is a possibility that the fauna may not have been completely recorded. Only 26 shells of *Burtoa* were recorded across all sites during the study so this species appears to occur at low density, thus potentially hindering detection when sample sizes are low. The minute *Afroguppya* sp. PT is also confined to the two Garusi sites. The Garusi sites were also situated on Mbugu black cotton soils (vertisols), which are unstable soils prone to large scale expansion and shrinkage and are thus perhaps less likely to support rich mollusc faunas. *Burtoa* is present as a fossil in both the Upper and Lower Laetolil Beds (Table 1).

While urocyclid slugs were not found by TWINSPAN to be an indicator species, the present study found urocyclid slug species only in woodland/bushland (four sites), and open habitats (one site). Among terrestrial molluscs, live slugs are notoriously difficult to sample effectively, especially on a single site visit, during the daytime and in dry weather conditions, when individuals are likely to be sheltering underground and very difficult to detect [33]. However, we did not detect any, or any slug plates, at the forest sites. Slug plates are common fossils throughout the Upper Laetolil Beds (Table 1).

It is clear that there remains a considerable amount of habitat heterogeneity within the woodland/bushland group, but further research would be required to explore how the mollusc faunas relate to such variation in habitat structure, anthropogenic disturbance and/or climate.

### Relationship with environmental variables

#### Local environmental variables

Species richness (Chao-1 estimator) remains strongly associated with the number of individuals collected (Table 9), and both richness and abundance are positively correlated with site elevation, tree canopy cover, tree height and size, and litter depth. This is consistent with the conclusion that richer and more abundant faunas are associated with higher elevation forest sites that have taller and denser tree canopies. Neither species richness nor abundance correlate significantly with soil pH.

**Table 9:** Pearson correlation coefficients between local environmental variables and Chao-1 species richness and abundance (no. individuals collected).

Variable	Abundance (individuals/collector)	P	Chao-1 species richness	P
**Chao-1 species richness**	0.781	0.001**	-	-
**No. individuals**	0.911	0.000**	-0.801	0.000**
**Soil pH**	0.289	0.296	0.333	0.225
**Elevation**	0.773	0.001**	0.531	0.041*
**Slope**	0.019	0.947	-0.055	0.845
**Litter depth**	0.591	0.020*	0.588	0.021*
**Canopy cover**	0.623	0.013*	0.677	0.006*
**Understorey cover**	0.382	0.160	0.503	0.056
**Tree height**	0.622	0.013*	0.733	0.002*
**Mean dbh**	0.529	0.042*	0.494	0.061
**Maximum dbh**	0.566	0.028*	0.518	0.048*
**Disturbance**	-0.224	0.422	0.127	0.653

**, P<0.001; *, P<0.05.

#### Wider environmental variables

Canonical Correspondence Analysis (CCA) was used to investigate the relationships between the snail faunas from the Laetoli and Mbulu sites and the wider environmental variables (the BIOCLIM variables, elevation and aridity index (AI_ET0)). There is a high degree of collinearity amongst the environmental variables, so care is needed when interpreting associations between individual variables and the faunas. Quantitative data are not available for the Mbulu sites so presence absence data were used in this analysis.

**Fig 5:**
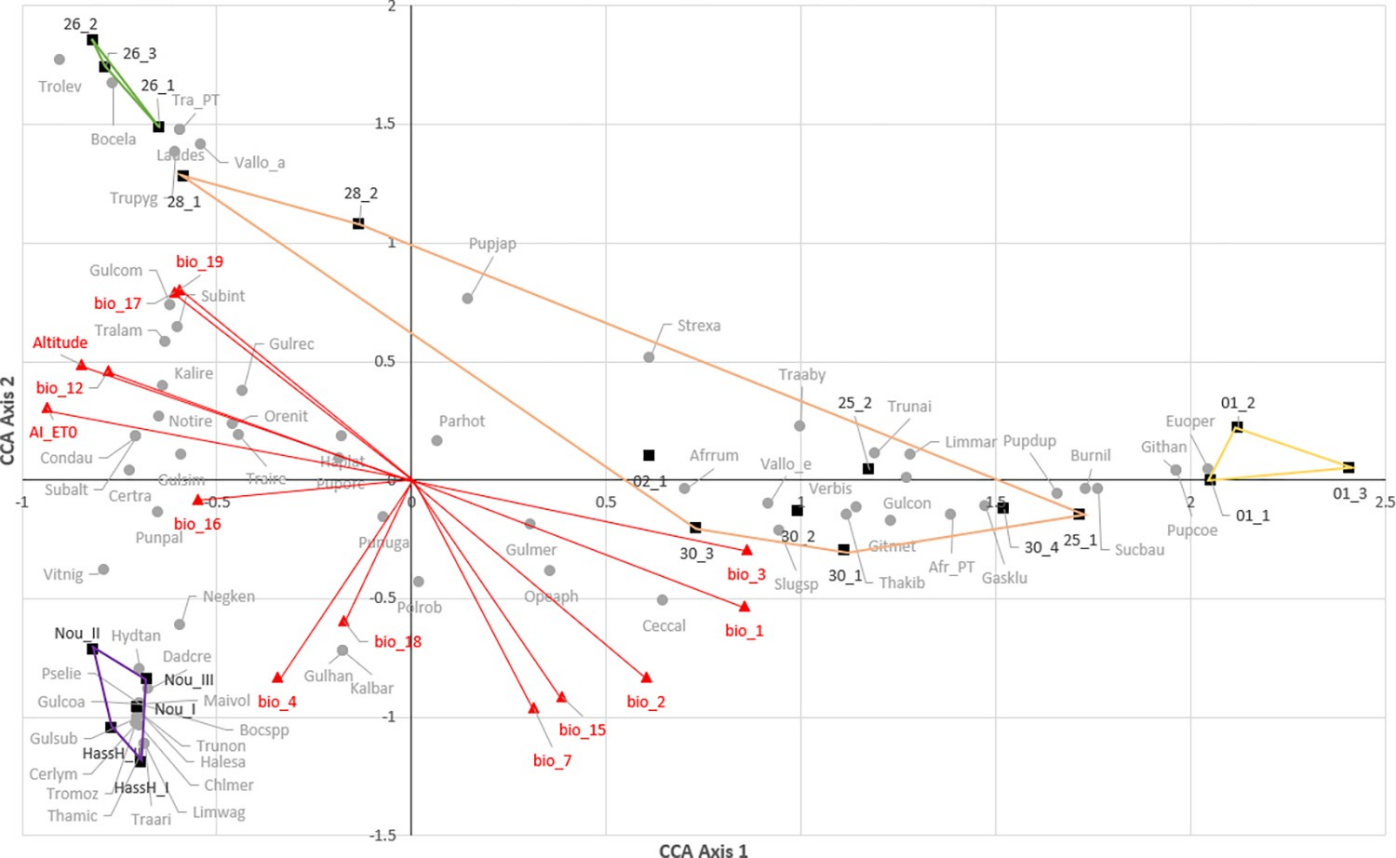
Canonical correspondence analysis (CCA) triplot of mollusc and environmental data from 15 sites at Laetoli and 5 forest sites on the Mbulu Plateau.

The first three axes account for 40%, 21% and 13% of the total variation respectively (Table 10). Axis 1 clearly arranges the sites along a strong habitat gradient extending from upland closed forest on Lemagurut at Laetoli and on the Mbulu plateau, through the intermediate riverine woodlands (28.I and 28.II) and the larger woodland/bushland group, to the open habitat types on the Osinoni plain (Fig 5).

**Table 10:** Eigenvalues and % variation accounted for by axes 1–5 of the CCA.

Axis	Eigenvalue	%
1	0.633	40.02
2	0.336	21.26
3	0.209	13.20
4	0.115	7.253
5	0.099	6.259

Axis 1 correlates negatively (Table 11) with the aridity index (AI_ETO), altitude and mean annual precipitation (BIO12), and positively with isothermality (BIO3) and annual mean temperature (BIO1). Thus, sites scoring highly on this axis tend to be situated at low elevation and experience higher and more variable temperatures regimes and relatively xeric conditions (ie low AI_ET0); clearly, these are conditions that would be expected in open habitats (e.g. grassland, xeric scrub).

**Table 11:** Correlation of the CCA axes with environmental variables.

Variable	Axis 1	Variable	Axis 2
AI_ETO	-0.935	BIO7 = Temperature Annual Range	-0.958
Altitude	-8.849	BIO15 = Precipitation Seasonality	-0.916
BIO12 = Annual Precipitation	-0.778	BIO2 = Mean Diurnal Range	-0.831
BIO17 = Precipitation of Driest Quarter	-0.610	BIO4 = Temperature Seasonality	-0.830
BIO19 = Precipitation of Coldest Quarter	-0.597	BIO18 = Precipitation of Warmest Quarter	-0.595
BIO16 = Precipitation of Wettest Quarter	-0.550	BIO1 = Annual Mean Temperature	-0.530
BIO4 = Temperature Seasonality	-0.345	BIO3 = Isothermality (BIO2/BIO7) (×100)	-0.295
BIO18 = Precipitation of Warmest Quarter	-0.175	BIO16 = Precipitation of Wettest Quarter	-0.082
BIO7 = Temperature Annual Range	0.313	AI_ETO	0.305
BIO15 = Precipitation Seasonality	0.386	BIO12 = Annual Precipitation	0.463
BIO2 = Mean Diurnal Range	0.602	Altitude	0.487
BIO1 = Annual Mean Temperature	0.854	BIO17 = Precipitation of Driest Quarter	0.792
BIO3 = Isothermality (BIO2/BIO7) (×100)	0.860	BIO19 = Precipitation of Coldest Quarter	0.802

**Fig 6:**
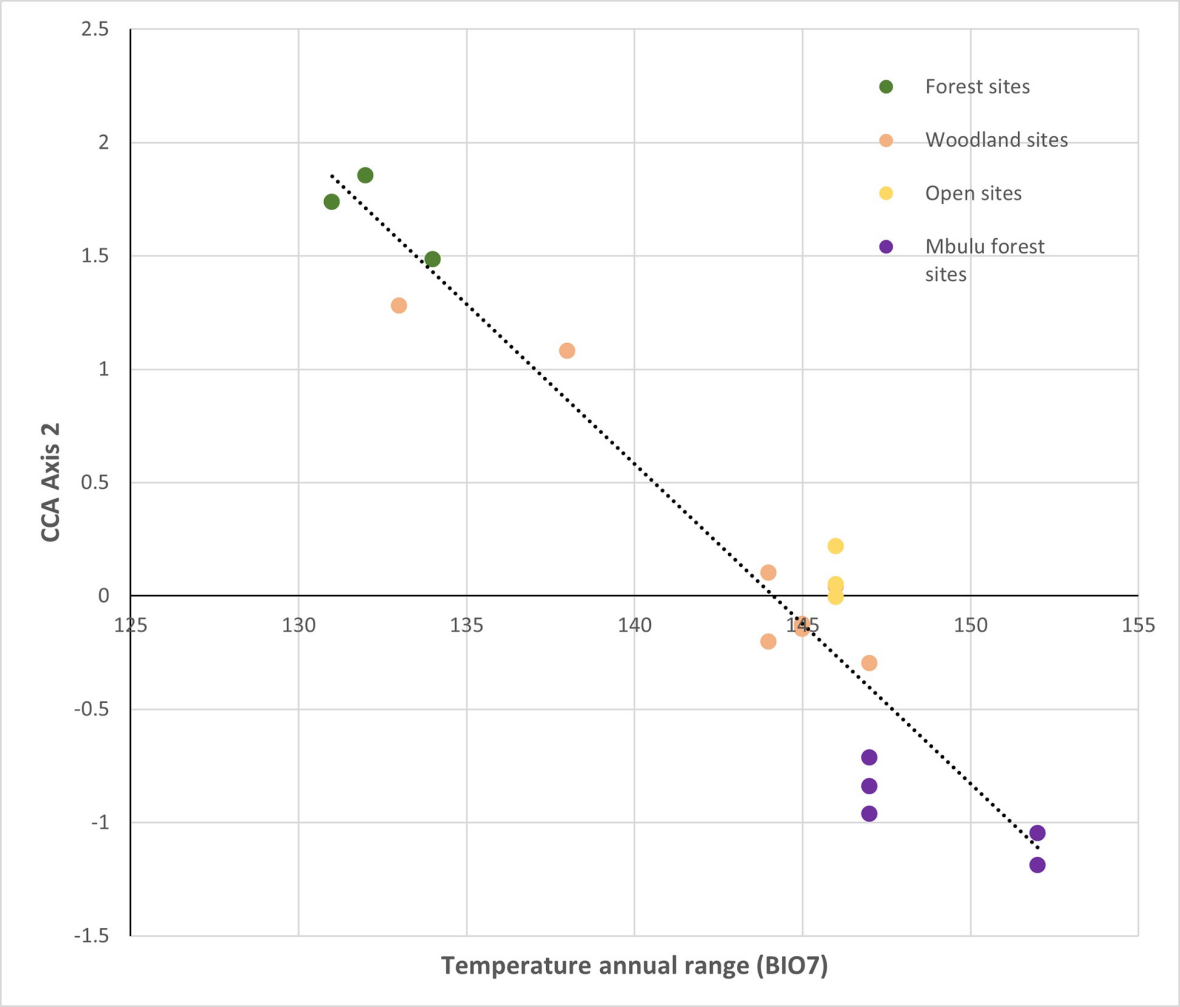
Relationship between CCA Axis 2 and temperature annual range (BIO7).

Axis 2 accounts for less variation but it appears to separate the Laetoli and Mbulu forest sites (Fig 5). It is most strongly related to the scale of variation in temperature and precipitation, at both diurnal and seasonal scales. It is negatively correlated with annual temperature range (BIO7) (Fig 6), precipitation seasonality (BIO15), and diurnal temperature range (BIO2) (Table 11), and positively with annual precipitation during the driest (BIO17) and coldest (BIO19) quarter (which, like several of the BIOCLIM variables, are very likely to be correlated). Thus, relative to the Lemugurut forest sites, forest sites on the Mbulu plateau tend to experience more strongly seasonal rainfall patterns, with lower rainfall levels during the coldest and driest months and greater variation in diurnal temperature. In contrast, Lemugurut’s forests experience a less strongly seasonal climate and less diurnal fluctuation, with a relatively low annual temp range, low precipitation seasonality, low diurnal temperature range and higher rainfall during the coldest and driest quarter of the year.

The analysis was then extended, using correspondence analysis (CA), to include the Lower and Upper Laetolil Beds and the Upper Ndolanya Beds (treating them as sites, without environmental data) and using only the eleven species with fossil equivalents (Table 12). As in Tattersfield [1], the Upper Laetolil Beds were subdivided into four stratigraphic subunits that allow the faunas to be arranged in a temporal sequence, from oldest to youngest, these being below Tuff 3, between Tuffs 3 and 5, between Tuffs 5 and 7, and between Tuff 7 and the Yellow Marker Tuff.

**Table 12:** Attribution of extant species to fossil species, and their distribution in the Laetoli fossil beds (fossil data from Table 1).

		Stratigraphic occurrences						
Fossil species	Extant species	Lower Laetolil Beds	Upper Laetolil Beds (all)	Upper Laetolil Beds (tuffs 0–3)	Upper Laetolil Beds (tuffs 3–5)	Upper Laetolil Beds (tuffs 5–7)	Upper Laetolil Beds (tuff 7-yellow marker tuff)	Upper Ndolanya Beds
		LLB [1,14]	ULB_all [1,19]	ULBT0_3	ULBT3_7	ULDT5_7	ULBT7_YMT	UNB
’*Succinea*’ sp. A	*Succinea baumanni*	1	1	0	0	0	0	0
*Burtoa nilotica*	*Burtoa nilotica giraudi*	1	1	1	0	1	1	0
*Limicolaria martensiana*	*Limicolaria martensiana catharia*	1	1	0	0	1	0	0
*Subuliniscus sp*. *A*	*Subuliniscus alticolus*	0	0	0	0	0	0	1
*Streptostele (Raffraya)* aff. *horei*	*Streptostele exasperata* (??)	0	1	0	0	1	1	1
*Gittenedouardia laetoliensis*	*Gittenedouardia metula*	1	1	1	1	1	1	0
*Cerastus* sp. A	*Cerastus trapezoidea masaica*	1	0	0	0	0	0	0
*Pupoides coenopictus*	*Pupoides coenopictus*	1	0	0	0	0	0	0
*Halolimnohelix rowsoni*	*Haplohelix lateaperta*	0	1	0	0	0	1	0
*Trochonanina* sp. B	*Trochonanina* cf. *levistriata*	1	1	1	1	1	1	0
Urocyclid slug indet.	Slug plate (gen. & sp. indet.)	0	1	1	1	1	1	1

**Fig 7:**
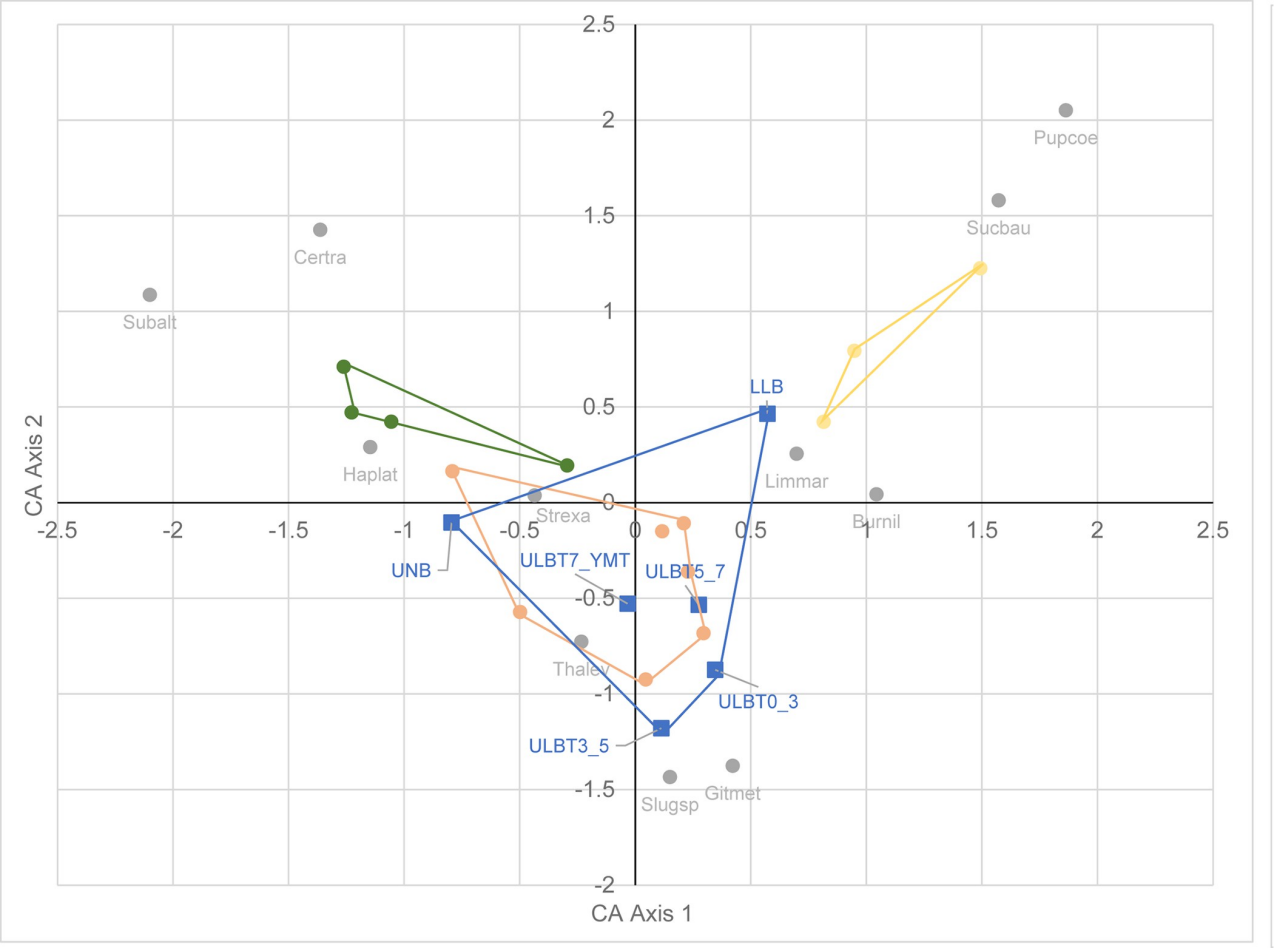
Correspondence analysis (Axis 2 versus Axis 1) of mollusc faunas from Laetoli recent sites and fossil beds, based on 11 species and their fossil equivalents.

The first two axes of the CA (Fig 7) account for 56% of the total variation and, as before, Axis 1 reflects a strong habitat gradient extending from closed forest to open conditions, and correlating strongly with AI_ET0, mean diurnal range (BIO2) and isothermality (BIO3). All the Upper Laetolil subunits lie within the central part of this continuum (Fig 7), occupying the region containing the woodland/bushland sites in this study. In contrast, on Axis 1, the Upper Ndolanya Beds lie closer to the forest sites and the Lower Laetolil Beds closer to the open habitats, thus suggesting the presence of more humid and more xeric conditions respectively. Axis 2, accounting for 22.9% of total variation, correlates weakly with precipitation seasonality (BIO15).

**Fig 8:**
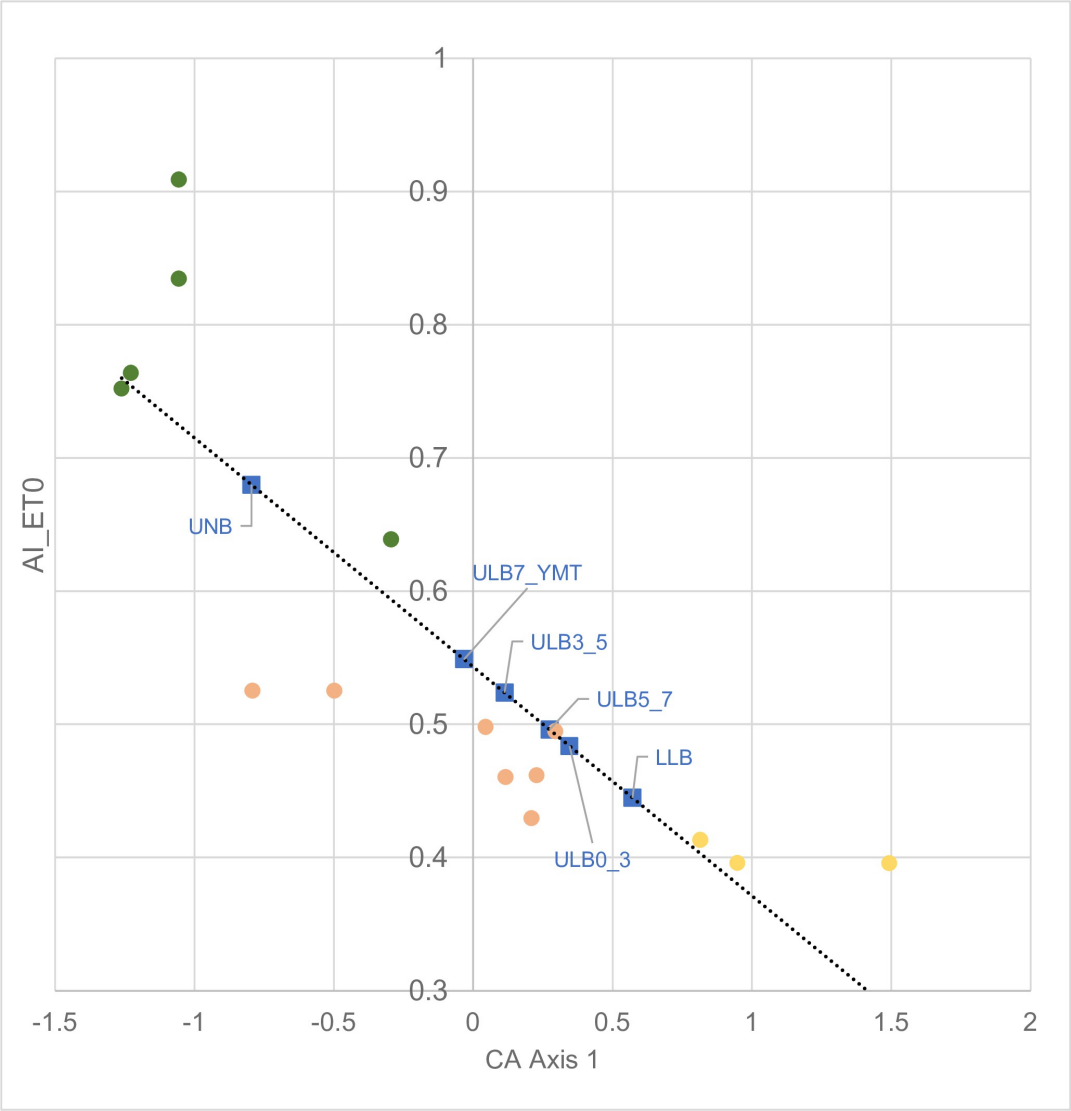
Relationship between CA Axis 1 and aridity index (AI_ET0).Based on 11 species and their fossil equivalents, to which Axis 1 weightings of faunas from the LLB, ULB and UNB stratigraphic units and subunits have been added. Horizontal lines show boundaries of climate categories based on Aridity Index values [42]. The regression line is highly significant r^2^ = 0.732 P<0.001.

Plotting Axis 1 scores for the fossil sites against AI_ET0 (Fig 8) and annual precipitation (BIO12), both strong correlates of Axis 1, allows an estimate of aridity index and annual precipitation based on their mollusc faunas (Table 13). By adopting the widely used UNEP climate classification [42], the values of AI_ET0 place the Upper Ndolanya and Lower Laetolil Beds in the Humid and Semi-arid categories respectively, with estimates for the four Lower Laetolil subunits lying in the Dry sub-humid or Semi-arid classes. Overall, this analysis suggests a trend for decreasing aridity and increasing humidity/precipitation from the oldest Lower Laetolil stratigraphic unit to the most recent Upper Ndolanya Beds. The exception to this pattern is the Upper Laetolil subunit bounded by Tuffs 5 and 7 where the results indicate a slight increase in aridity relative to the immediately preceding subunit (ie between Tuffs 3 and 5). However, these observations are necessarily tentative because the total estimated range in annual rainfall, across all four Upper Laetolil subunits, is only 33mm/yr. These estimates place estimated mean annual precipitation during the Pliocene towards the upper end of the current level reported from the Laetoli area of 700–900 mm. In terms of the study sites, BIO12 gives MAP figures of 1008–1088 mm/yr for the Lemugurut forest sites, 854–859 mm/yr for the woodland/bushland sites and 839 mm/yr for the Osinoni open sites.

**Table 13:** Estimates of Mean Annual Precipitation (MAP, mm/yr) and Aridity Index (AI_ET0 x 10000) based on fossil mollusc faunas recorded from six stratigraphic units/subunits.

Stratigraphic Unit/Subunit	Estimated MAP (mm/yr)	Estimated Aridity Index
Lower Laetolil Beds	847	4400
Upper Laetolil Beds (ULB), below Tuff 3	866	4800
ULB, between Tuffs 3 and 5	887	5200
ULB, between Tuffs 5 and75	873	5000
ULB, between Tuff 7 and Yellow Marker Tuff	899	5500
Upper Ndolanya Beds	965	6800

#### Faunal overlap with nearby areas

**Fig 9:**
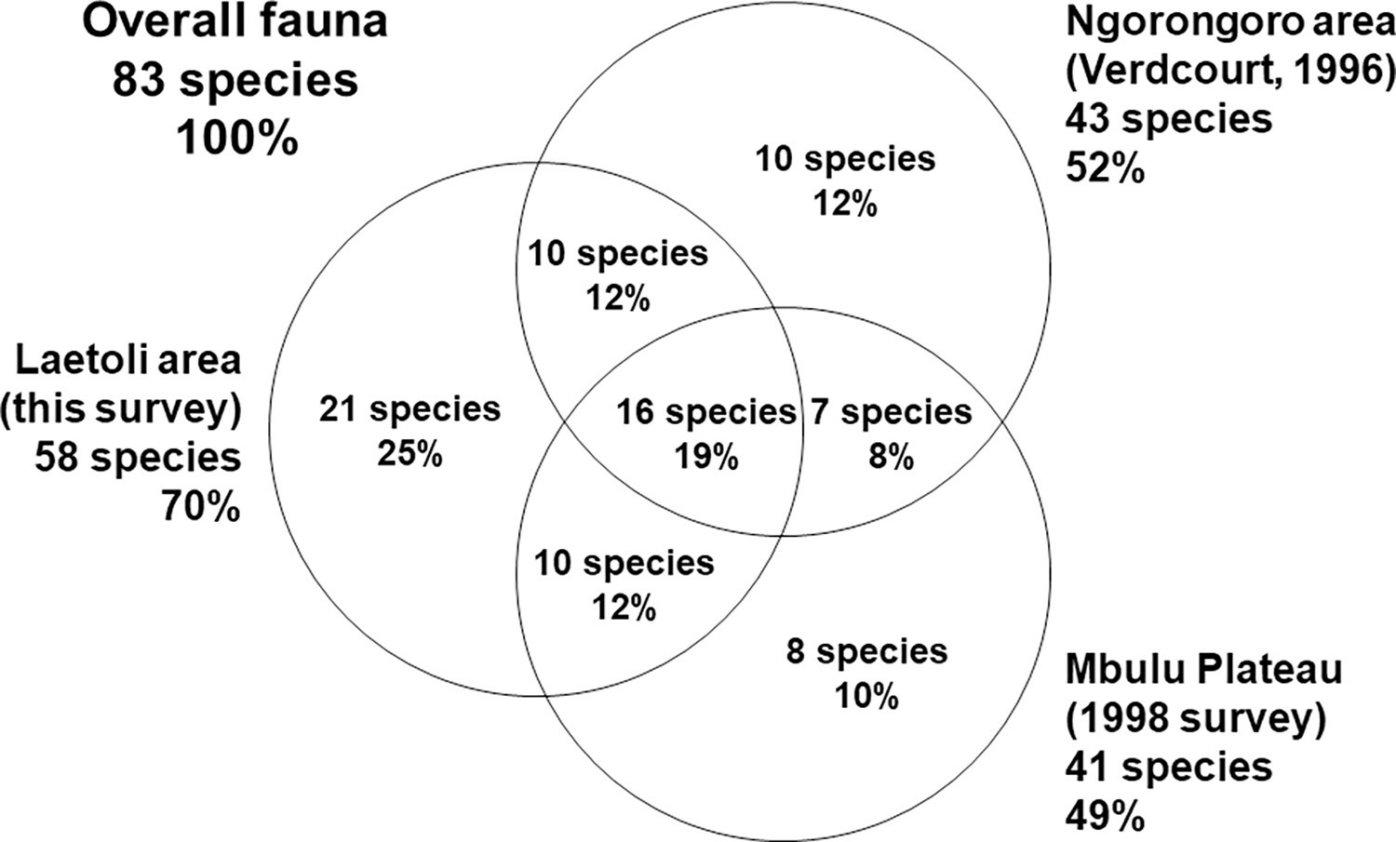
Overlaps with the mollusc faunas of Ngorongoro and the Mbulu Plateau.

To put the Laetoli data in a regional context, making the most of all available mollusc data, and to account for species that have changed their distributions since the Pliocene, faunal lists were compared with those for the Ngorongoro area and for the forests on the Mbulu Plateau. The total pool of species currently recorded from these areas stands at 83 (including the five probably undescribed species from Laetoli). The 58 species found at Laetoli represent only 70% of those in the region. There appears to be variation across the region (Fig 9), such that only 16 (19%) of the species have been reported from all three areas, and a total of 39 species (47%) have only been found in one of the three. Thus, the apparent absence from Laetoli of some taxa present as fossils could reflect historical regional changes.

A total of 26 species (31% of all those in the region) have been recorded from both Laetoli and Ngorongoro. Verdcourt’s [27] paper on the molluscs of Ngorongoro was a compilation of records from the literature and casual collecting in the 1960s and 1970s. It included a few records from the Serengeti area, but the majority were from forest habitats at 1800–2250 m on the Crater Highlands between Oldeani and Empakai. The list reports 77 terrestrial taxa in total but (as is characteristic of Verdcourt’s faunistic work) at least 29 are incompletely identified forms (e.g. “sp.”, “var.”, “juv.” etc.) some of which are probably part of the normal variation of the taxa named in his, or our, list. The more taxonomically conservative value of 43 species has been used here.

The few species that Verdcourt [27] recorded from non-forest habitats accord with our own findings at Laetoli. These species include *Succinea baumanni* “abundant on seasonally wet open ground”; *Burtoa nilotica giraudi* “in *Acacia seyal* stands”; *Limicolaria martensiana* “Serengeti, Naguro [;] Seronera, Naabe Hill [and] Empakaai, along trunk road at rain gauge in moorland”; *Atoxon hildebrandti* “Laetoli Gorge […] Serengeti Plains, Seronera”.

Most notable among the forest taxa reported by Verdcourt [27], that were not found in the present survey, is *Pseudoglessula*, a robust and easily recognisable genus present in the Upper Laetolil Beds. Verdcourt [26] lists *P*. *monticula*, *P*. *liederi* and two other *Pseudoglessula* morphospecies from Ngorongoro. Smaller taxa not found in the present surveys include *Maizania volkensi*, *Trachycystis ariel* and *Afroconulus iredalei*, all forest species occurring widely in the volcanic highlands of Tanzania and Kenya.

A total of 26 species (31% of those in the region) have been found at both Laetoli and on the Mbulu Plateau. The Mbulu sites share 23 species (about 27% of all those in the region) with Ngorongoro, including *P*. *liederi*, *M*. *volkensi*, *T*. *ariel*, and *A*. *iredalei*. This indicates that much of the Ngorongoro forest fauna extends to the south of Lake Eyasi. Other taxa do not appear to extend to Mbulu, e.g. the achatinid *Liobocageia elata*, which has been found only at Ngorongoro and Laetoli (Lemagurut). The most similar material from Mbulu is much more slender, a phenomenon also observed at Oldeani and Empakaai [27]. *Liobocageia elata* may be one of the few narrow-range endemics in the fauna. As the other East African species of *Bocageia/Liobocageia* live on Mt. Elgon and Mt. Rwenzori, *B*. *elata* is either an ancient relict or in fact an endemic belonging to another genus, perhaps *Oreohomorus*, *Subuliniscus* or even *Kenyaella*. It exemplifies a trend for high endemism among forest molluscs in East Africa. Other narrow-range endemics in the region’s fauna include *Gulella ngorongoroensis* (found in all three parts of the region) and *Dadagulella cresswelli* (found at Mbulu, and more recently at Ngorongoro [43]).

The majority of the 31 species found at Laetoli, but not reported from Ngorongoro by Verdcourt [27], are small taxa that are among the most likely to have been overlooked by casual collectors. These include the five probably undescribed species. Most of those from forest or woodland also occur on the Mbulu Plateau, e.g. *Negulus kenianus*, *Punctum ugandanum* and *Silvigulella hanangi* and so may range throughout the region but so far have been missed at Ngorongoro. Conversely, the absence of *Macroptychia*, *Gastrocopta*, *Vertigo (*formerly *Afripupa)*, *Pupilla* and *Pupoides* from both Verdcourt’s [27] list and the Mbulu Plateau Forest surveys may instead reflect their avoidance of forest habitats. If correct, this would suggest these differences are consistent across this region of Tanzania.

At Olduvai Gorge, only a small number of terrestrial mollusc species (10) have been recorded. One species each of *Streptostele*, *“Homorus*,” and urocyclid slug have been recorded in the Late Pliocene beds and are considered to support a woodland habitat, while *Limicolaria* and *Achatina* appear in the Pleistocene beds [23]. Recent, unpublished work also yielded *Succinea*, *Gittenedouardia*, *Euonyma* and *Haplohelix* from the Holocene layers, which are thought to reflect a relatively open bushland similar to Olduvai today (B Rowson, unpublished 2017).

## Discussion

This study describes land-snail faunas from several strongly contrasting vegetation types ranging from Lemugurut’s afromontane rainforest, through a rather disparate group of woodland/bushland sites associated with vegetation mosaics, to open grasslands and xeric scrub on the Osinoni plains. It represents one of the first studies to examine systematically the associations between land-snail faunas and vegetation type within a geographically restricted part of East Africa.

Significant differences were found between the mollusc faunas of different habitat types (Table 5), in species composition, richness and abundance (Tables 4–6, Figs 4 and 5). That total mollusc species richness was highest in woodland/bushland habitats, rather than in the afromontane forests on Lemugurut, is somewhat unexpected because other studies have typically reported lower species richness in savanna than adjacent areas of forest [12,13,44]. (However, this may, in part, reflect the small number of sites (ie three) sampled on Lemugurut and the inclusion of the riverine forest sites 26.I and 26.II, which contain a mixture of forest and non-forest species, in the non-forest group). As far as is known, there are no comparable detailed studies on the land-snail faunas of xeric grasslands and xeric scrub.

Previous studies on the composition and structure of East African land-snail faunas have focussed on forest habitats, which are believed to support most of the region’s molluscan diversity [2]. Most have dealt with elevational gradients (e.g. Uganda [5]; Mt Kenya [4]; Mwanihana, Tanzania [45]) or examined localised variation in faunas within forest habitats (e.g. [10,46,47]). But in some cases, the focus has been on historical or biogeographical factors [5,45], and this has identified variation and/or discontinuities that cannot be readily accounted for by current environmental conditions. The only detailed ecological study [12] of land-snails associated with an East African savanna ecosystem (in Rwanda) focussed on landscape scale factors and concluded that mollusc species richness was most strongly affected by distance from the remnants of rainforest occurring on an adjacent escarpment, with the secondary effects of annual precipitation (as reported here) and distance from the closest watercourse (or ‘next stream’). Wronski et al. [12] hypothesised that the savanna snail fauna’s persistence was dependent, in part, on spillover from adjacent rainforests. The present study did not examine landscape scale effects *per se*, but it did reveal strong compositional similarities between the faunas of the riverine forest sites (26.I and 26.II) and Afromontane forest fauna on Lemagurut, the former essentially being an impoverished version of the latter.

### Environmental variables and neighbouring faunas

Variation in precipitation (and correlates such as aridity levels) has been implicated in the structuring of East African land-snail forest faunas in other studies [4], and this project draws a similar conclusion across the broad range of vegetation types studied. At a more local level, and as reported in other studies [6,47], forest structure appears to play a role, and disturbance effects may also be apparent, as exemplified by the presence of the synanthropic achatinid *Limicolaria* when occurring in forest habitats.

Extension of the geographical scale to include forest sites on the Mbulu plateau suggests that seasonality and also possibly diurnal temperature variation (Fig 5) may be having a secondary effect. Thus, whilst Axis 1 of the CCA ordination clearly reflects major variation in precipitation/aridity, Axis 2 suggests that differences in the Lemugurut and Mbulu forest faunas may be associated with differences in climatic variability throughout the year (i.e. seasonality), the Lemugurut forests experiencing less seasonal patterns of precipitation, and receiving more rainfall during the coldest and driest months than those on the Mbulu plateau. Lemagurut forests also experience less diurnal temperature variation.

Historical biogeographic effects may also be relevant to the makeup of the Laetoli fauna. For example, the Mbulu fauna includes ‘western’ species that were not recorded at Laetoli (e.g. *Thapsia cf*. *microleuca*), whose distribution is mainly associated with Congolian rainforest habitats. Both Mbulu and Ngorongoro host *Pseudoglessula*, which we did not find at Laetoli despite it being known there as a fossil. None of the faunas have extant *Kenyaella*, which now appears to be restricted to central Kenya and eastern Tanzania [1,26,48]. Thus, whilst there is a large pool of local species, it remains possible that the absence of certain taxa present at Laetoli as fossils is due to past range changes, rather than current climatic differences. A complete mollusc fauna from any of the Laetoli beds has yet to be found living nearby.

### Sampling considerations

Alternatively, the apparent absence of certain moderately large and robust taxa may be due to their being restricted to habitats or localised areas at Laetoli that were not sampled in this study. However, we attempted to minimise this by sampling a wide range of habitat types and incorporating as much previously published evidence as possible. Judging by the small differences between our species counts and the Chao-1 estimator (Table 4), our list for each different habitat type is relatively complete. The very large and durable shells of (even juvenile) achatinids are less likely to have been overlooked, although the animals are strong crawlers and capable of accessing deep refuges. Sampling inefficiency is well-known to be a problem for slug inventory, given their exceptional ability to hide in soil cracks. Yet the complete lack of slugs or even slug plates from our forest samples, taken in humid conditions, may reflect a genuine difference. Tattersfield [1] placed some weight on the presence and relative abundance of slugs when interpreting the palaeoenvironments associated with the Laetoli fossil beds. He suggested that the abundance of slug plates recorded in the Upper Laetolil and Upper Ndolanya Beds might be indicative of a humid and possibly treed or forested environment, whereas the near absence of slugs in the Lower Laetolil Beds were interpreted as indicating more arid conditions. The East African fauna includes many urocyclid slug species, and, although their ecological associations are not known in detail, some species are known to live in forest habitats (e.g. [49]). However, the finding reported in this study, that slugs were absent from the forest sites and they were most frequent and abundant in the open and woodland/bushland habitats, means that Tattersfield’s [1] interpretation requires revision.

The high diversity of small snail species (under 10 mm long) in our study is typical of many extant East African faunas, though not of the Laetoli fossil faunas. This does not appear to be a sampling issue associated with the fossil fauna, so the relative rarity of small species in the beds remains to be explained. It is noted that other Miocene and Plio-Pleistocene fossil sites in East Africa have yielded quite rich micro-gastropod faunas [14], although the short list of Pliocene and Pleistocene taxa from Olduvai [23] resembles that of Laetoli in consisting mainly of large species.

### Value as indicator species and for palaeoecological reconstruction

Interpretation of Pliocene environments using fossil gastropods relies on the identification of appropriate extant analogue species and an understanding of the habitats and ecologies of these species, thus enabling inferences to be made about the likely Pliocene environment. This study has yielded further information about the mollusc faunas currently associated with the main vegetation types in the Laetoli-Endulen area. It facilitates the identification of appropriate surrogates and allows their value as environmental markers or indicators to be assessed with greater confidence, thus enabling further interpretation of the Pliocene environment.

Table 14 identifies putative analogues for all the Pliocene fossil taxa (listed in Table 1), and summarises their potential value as habitat indicators. It also shows the distribution of the Pliocene fossils in three major stratigraphic units (from [1])–the Lower and Upper Laetolil Beds, and the Upper Ndolanya Beds. The indicator taxa in Table 14 have been rearranged to illustrate the main differences in the three stratigraphic units, with species assessed to hold indicator value grouped together at the top of the table.

**Table 14:** Pliocene fossil taxa with their extant analogues, their potential value as habitat indicators, and their stratigraphic occurrences. Occurrences are from [1] with the record of *Pupoides coenopictus* from [14]. The Upper Laetolil Beds are here treated as a single unit.

	Pliocene fossil species	Extant analogue species	Habitat association and comments	Lower Laetolil Beds	Upper Laetolil Beds	Upper Ndolanya Beds
**I. Species considered strong habitat indicators**						
	’*Succinea*’ sp. A	*"Succinea" baumanni*	Restricted to open, arid to semi-arid conditions, perhaps containing areas of seasonal inundation.	**1**	**0**	**0**
	*Pupoides coenopictus*	*Pupoides coenopictus*	Restricted to open, xeric habitats.	**+**	0	0
	*Gittenedouardia laetoliensis*	*Gittenedouardia metula / hanningtoni*	Woodland/bushland, or more open conditions. Two extant species of *Gittenedouardia* were recorded during the study. In terms of shell shape, the fossil species matches *metula* more closely than *hanningtoni*, but both species avoid closed forest, being strongly associated with either woodland/bushland (*metula*) or open habitats (*hanningtoni*).	**3**	**131**	**0**
	*Burtoa nilotica*	*Burtoa nilotica giraudi*	Woodland/bushland and open habitats only; avoids closed forest.	**23**	**54**	**0**
	*Subuliniscus* sp. A	*Subuliniscus alticolus*	Restricted to closed, typically highland, forest habitat.	**0**	**0**	**11**
**II. Species considered moderately strong habitat indicators**						
	*Pseudoglessula (Kempioconcha)* aff. *gibbonsi*	None in present study	Subgenus *Kempioconcha* typically in woodland/bushland habitats; *P*. *(K*.*) liederi* is present in forest at Mbulu, and is recorded from Ngorongoro (Verdcourt, 1996).	**0**	**64**	**0**
	*Subulona pseudinvoluta*	None in present study	Typically closed forest, but also in more open forest types near the coast.	**0**	**231**	**0**
	*Kenyaella leakeyi*, *K*. *harrisoni*	None in present study	*Kenyaella* species are associated with closed and gallery forest habitats elsewhere in East Africa.	**0**	**905**	**49**
**III. Species considered weak habitat indicators**						
	Urocyclid slug indet.	Urocyclid slugs (all specimens)	Found only in woodland/bushland in this study, but genus/species unknown. Urocyclid slugs are common in forest elsewhere in East Africa.	**0**	**3328**	**506**
	*Streptostele (Raffraya)* aff. *horei*	*Streptostele exasperata*	Probably woodland/bushland; also cloud forest above semi-arid areas.	**0**	**12**	**3**
	*Cerastus* sp. A	*Cerastus trapezoidea masaicus*	Closed forest. Generic assignment of fossil material tentative (Tattersfield, 2011).	**3**	**0**	**0**
	*Halolimnohelix rowsoni*	*Haplohelix lateaperta*	Forest or woodland/bushland; not open habitats. Generic assignment of fossil material tentative (Tattersfield, 2011), but all East African Helicoidea live in shaded habitats.	**0**	**4**	**0**
**III. Species with no clear habitat assocation**						
	*Limicolaria martensiana*	*Limicolaria martensiana catharia*	No strong affinity—recorded in open, woodland/bushland and disturbed forest. Possibly indicator of disturbed conditions when in forest.	**168**	**1**	**0**
	*Trochonanina* sp. A, *T*. sp. B	*Trochonanina* cf. *levistriata*	None. Verdcourt (1987) suggested *T*. sp. B to be allied to *T*. *elatior*, which is a widesprerad species typically associated with bushland, savanna, Miombo woodland and grassland habitats. Unlike most species of *Trochonanina*, the species recorded in the study (*T*. cf. *levistriata*) is associated with upland closed forest.	**14**	**84**	**2**
	*Streptostele* sp. A	None in study	Unknown. Fossil material insufficient.	**0**	**1**	**0**
	*Gulella* sp. A	None in study	Unknown. Fossil material insufficient to assign species or section in this very speciose group.	**0**	**2**	**0**
	*Achatina (Lissachatina) zanzibarica*, *A*. *(L*.*) fulica hamillei*	None in study	*Lissachatina* may be present in a wide range of habitats. Habitat differences between the two species reported as fossils need further study.	**80**	**136**	**0**
	Indeterminate	n/a	n/a	**2**	**13**	**3**
			Total number of gastropod specimens (excluding slugs)	**294**	**1638**	**68**
			Total number of gastropod specimens (including slugs)	**294**	**4966**	**574**

The Lower Laetolil beds is the only unit in which the strong indicators of open habitats, ‘*Succinea’* sp. and *Pupoides coenopictus* (record from [14]), have been found. This suggests that this stratigraphic unit (or perhaps parts of it since it spans a long time period, see below) was associated with a semi-arid environment, probably with open grassland or/and xeric scrub making a significant contribution. Species in the circumglobal family Succineidae are typically associated with damp habitats such as marshes, and Tattersfield [1] suggested that the presence of ’*Succinea’* sp. in the Lower Laetolil Beds might ‘indicate damper conditions, at least locally or during some periods of time’. However, this study has demonstrated that *Succinea* has a strong affinity for open and relatively xeric habitats at Laetoli, although it is likely that these areas are prone to short term flooding during the rainy season. Only one *Succinea* fossil was recovered [1], and the *Pupoides* record relies on Pickford’s [14] account. This conclusion is therefore necessarily tentative, although it is not inconsistent with the interpretation of the Lower Laetolil, or the correspondence analysis reported here (Figs 7 and 8), as a relatively xeric and open environment possibly also containing patches of bushland and/or woodland [1]. The fauna of the Lower Laetolil Beds is dominated by large-shelled achatinids (*Burtoa*, *Limicolaria and Achatina*), with *Gittenedouardia* also present at lower frequency. Both *Burtoa* and *Gittenedouardia* are recognised here as non-forest indicators, thus lending further support to an interpretation of the Lower Laetolil as a largely open environment. However, the Lower Laetolil Beds span a period of at least 0.5myrs so some temporal variation seems very likely [50]. A small number of shells tentatively identified by Tattersfield [1] as a *Cerastua* species, which is regarded here as a weak indicator of closed forest conditions, might reflect temporal variation in conditions, or possibly the occurrence of patches of forest, during the Lower Laetolil. Some of these genera were also recorded in a savanna ecosystem in Rwanda [12], in particular, *Succinea* (*princei* Preston), although only as 2 specimens, and two *Gittenedouardia* species, one recorded in moderately large numbers. However, [12] did not find *Burtoa*, despite Rwanda being closer to the centre of *Burtoa*’s current geographical range, and Laetoli being on or close to the edge.

The Upper Laetolil Beds lacks open habitat indicators, but includes non-forest indicators (*Burtoa* and *Gittenedouardia)*, and forest indicators (*Kenyaella and Halolimnohelix*, neither of which was found during the current survey, but whose preferences are well-established elsewhere [1,14]. Of the extant taxa we found, the species that most closely resembles *Kenyaella* in appearance is *Liobocageia elata*; it too was confined to forest habitats. This mixture of indicators suggests a mosaic of vegetation types, including elements of forest (perhaps gallery forest) among woodland and bushland in a grassland matrix. The presence of urocyclid slugs, which we did not record from forests, in the Upper Laetoli Beds is not inconsistent with this. A mosaic of forest, woodland and bushland habitats is also consistent with previous habitat interpretations (reviewed in [21]) and the proposition that the range of vegetation types in the Upper Laetolil was similar to that seen today in the area [25]. We estimated annual rainfall in the Upper Laetolil as 866–899 mm/yr (Table 13), which is slightly higher than currently received by the woodland/bushland areas in the Laetoli-Endulen area (854-859mm/yr). The difference is small so conclusions are tentative, but it may indicate that the area experienced a slightly more mesic climate, perhaps resulting a more wooded/forested environment, during at least some periods in the Upper Laetolil.

The Upper Ndolanya Beds have only three species assessed to hold indicator value, out of the five recorded. These comprise one strong (*Subuliniscus*) and one moderate (*Kenyaella*) forest indicator, and *Streptostele* aff. *horei* which is assessed as a weak woodland/bushland species. Based on this, it may be inferred that the UNB experienced more mesic, or even humid, conditions than either the Upper or Lower Laetolil Beds, and that it probably contained areas of closed woodland and/or forest. This conclusion is supported by our annual precipitation estimate of 965mm/yr (Table 13), which would assign the Upper Ndolanya to UNEP’s Humid climate type [42]. This conclusion needs to be qualified because the small total number of shells recovered from the Upper Ndolanya Beds hinders interpretation. In general, forested habitats across East Africa are often associated with richer and more abundant mollusc faunas (although in this study more species were recorded in the woodland/bushland group than in the forest), so the low richness (4 determined species) and scarcity (68 specimens) is not entirely consistent with this conclusion.

Several independent lines of evidence present a conflicting picture of the palaeoecology of the UNB, and of the transition from the earlier ULB [22]. The consensus view, based mainly on evidence from large mammals, is that landscapes of both the ULB and UNB contained a mosaic of woodland, grassland and shrubland, but that there was a climatic and ecological shift to drier conditions in the UNB that led to an increase in the proportion of grassland at the expense of the woodland and shrubland elements in the mosaic. However, our interpretation lends support to inferences obtained from ostrich eggshells and rodents, which suggest a transition to more mesic conditions in the UNB and a landscape more dominated by woodland and/or forest. Harrison [22] suggests that it may be possible to reconcile these differences by recognising that the different lines of evidence offer insights into the paleoecology of Laetoli at different spatial scales. Thus, evidence from large mammals is of relevance to the wider Laetoli landscape, whereas ostrich shells, snails and rodents tend to have much more restricted ranges and more focussed habitat requirements.

Finally, our analyses produce a refined estimation of climatic conditions at Pliocene Laetoli, based on the study of the extant mollusc fauna. This suggested that annual precipitation ranged from 847–965 mm/yr. These values lie within the range suggested by Verdcourt [19, p. 450], who estimated rainfall of ‘625–1000 mm’ and commented that ‘if the evergreen forest was narrow and supported by a permanent river then it could have been near the lower level; many of the species could not, however, have existed in grassland with scattered trees nor in woodland." However, it is possible that Verdcourt later realised that may have been overstated, once it was appreciated that slugs can live in quite dry habitats. Our estimated Pliocene precipitation, across all stratigraphic across units, spans a narrower range than Verdcourt’s but it is similar or perhaps slightly higher than that experienced by Laetoli today. Andrews et al. [25] provide a figure of 700–900 mm per year across the area, and the Worldclim database [38] (BIO12) returns 839–859 mm per year for the open and woodland/bushland sites in our study, and 839–1088 mm/yr across all the sites we studied. Other factors are also likely to be important influences on the vegetation but, as illustrated by the variation in the aridity index and assignment to UNEP climate classes, such differences in rainfall can have very significant influences on vegetation.

## Supporting information

S1 Table Species x sites dataset matrix. (XLSX)
